# The evolution of strand preference in simulated RNA replicators with strand displacement: Implications for the origin of transcription

**DOI:** 10.1186/1745-6150-3-33

**Published:** 2008-08-11

**Authors:** Nobuto Takeuchi, Laura Salazar, Anthony M Poole, Paulien Hogeweg

**Affiliations:** 1 Theoretical Biology and Bioinformatics Group, Utrecht University, Padualaan 8, 3584 CH, Utrecht, The Netherlands; 2 Department of Molecular Biology and Functional Genomics, Stockholm University, SE-10691, Stockholm, Sweden; 3 School of Biological Sciences, University of Canterbury, Private Bag 4800, Christchurch, New Zealand

## Abstract

**Background:**

The simplest conceivable example of evolving systems is RNA molecules that can replicate themselves. Since replication produces a new RNA strand complementary to a template, all templates would eventually become double-stranded and, hence, become unavailable for replication. Thus the problem of how to separate the two strands is considered a major issue for the early evolution of self-replicating RNA. One biologically plausible way to copy a double-stranded RNA is to displace a preexisting strand by a newly synthesized strand. Such copying can in principle be initiated from either the (+) or (-) strand of a double-stranded RNA. Assuming that only one of them, say (+), can act as replicase when single-stranded, strand displacement produces a new replicase if the (-) strand is the template. If, however, the (+) strand is the template, it produces a new template (but no replicase). Modern transcription exhibits extreme strand preference wherein anti-sense strands are always the template. Likewise, replication by strand displacement seems optimal if it also exhibits extreme strand preference wherein (-) strands are always the template, favoring replicase production. Here we investigate whether such strand preference can evolve in a simple RNA replicator system with strand displacement.

**Results:**

We first studied a simple mathematical model of the replicator dynamics. Our results indicated that if the system is well-mixed, there is no selective force acting upon strand preference per se. Next, we studied an individual-based simulation model to investigate the evolution of strand preference under finite diffusion. Interestingly, the results showed that selective forces "emerge" because of finite diffusion. Strikingly, the direction of the strand preference that evolves [i.e. (+) or (-) strand excess] is a complex non-monotonic function of the diffusion intensity. The mechanism underlying this behavior is elucidated. Furthermore, a speciation-like phenomenon is observed under certain conditions: two extreme replication strategies, namely replicase producers and template producers, emerge and coexist among competing replicators.

**Conclusion:**

Finite diffusion enables the evolution of strand preference, the direction of which is a non-monotonic function of the diffusion intensity. By identifying the conditions under which strand preference evolves, this study provides an insight into how a rudimentary transcription-like pattern might have emerged in an RNA-based replicator system.

**Reviewers:**

This article was reviewed by Eugene V Koonin, Rob Kinght and István Scheuring (nominated by David H Ardell). For the full reviews, please go to the Reviewers' comments section.

## Background

It is often pointed out that the simplest conceivable examples of a self-replicating evolving system, i.e. a replicase RNA which can copy itself, would be an example of dual genotype and phenotype, in that it is both replicator and its own template. The expectation is thus that the distinction between genotype and phenotype "evolved" at some later stage. However, assuming that replication is broadly similar to modern systems (proceeding 5' → 3', and reading 3' → 5' from a template), a second strand will always be synthesized as complementary to a template. Since all substrates will rapidly become double-stranded and, by default, become unavailable for replication, the problem of how to separate the two strands has been considered a major issue for the early evolution of self-replicating RNA [1,2] (see refs. [3-9] for the population biological consequence of the growth limitation due to double-strand formation).

**Figure 1:**
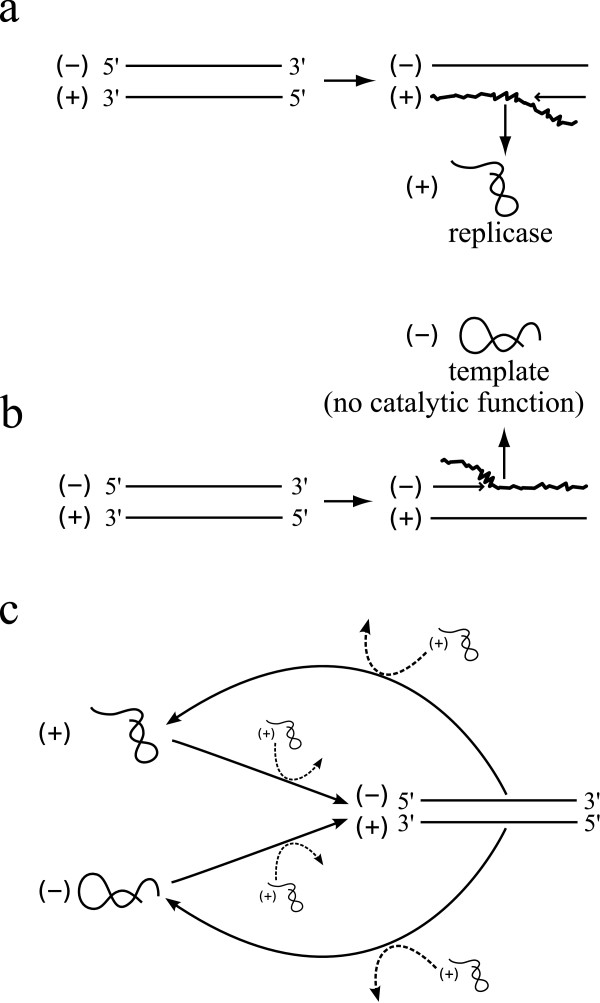
**A scheme of self-replicating RNA with strand displacement**. **a **and **b **show the two possible outcomes for replication from a double-stranded RNA by strand displacement. The single-stranded (+) catalyzes replication reactions, and is thus replicase. The single-stranded (-) carries no catalytic function. While it is arbitrary whether we designate single-stranded (+) strands or single-stranded (-) strands as replicases, the assumption that only one of the strands is the replicase is important. **c **shows the entire set of self-replication processes. Solid arrows represent replication reactions, where the origin of the arrows is the template and the end point of the affows is the product of replication. Dashed arrows represent catalysis. Note that both single-stranded (+) and (-) can serve as a template for replication, wherein replication gives rise to a double-stranded molecule.

The expectation among those working on experimental self-replicating RNA is that such a double-stranded intermediate could be copied by strand displacement (as in some RNA viruses) [1,10]. Copying by strand displacement permits either strand of a double-stranded RNA to serve as template. In Fig. 1a, the (+) strand codes for a replicase capable of template-directed RNA polymerization. Its complement, the (-) strand, has no catalytic function. Copying by strand displacement produces two possible outcomes, depending upon which template is utilized. Where the (-) strand is the template, copying with strand displacement will result in a new (+) strand being synthesized, the displaced (+) strand being able to fold up into an active (+) replicase (Fig. 1a). The net outcome is thus production of a new replicase molecule. However, if the (+) strand is template, the net outcome is production of a new copy of the (-) strand, the displaced strand folding up, but possessing no function (Fig. 1b). Hence, if the replicase exhibits no preference for either end of the double-stranded template, these two molecules will be produced in about equal amounts; only 50% of copying events will yield new replicases. In contrast, modern transcription systems exhibit extreme strand preference, in that (-) strands (anti-sense strands) are always the template. Likewise, replication by strand displacement seems optimal if it also exhibits such extreme strand preference favoring replicase production [always using (-) strands as a template].

Here we ask whether such preference towards producing single-stranded (+) replicases can actually evolve, and hence, whether a rudimentary transcription-like pattern can emerge in a simple RNA-based replicator system with strand displacement. To address this question, we first investigate a very simple mathematical model for a system of self-replicating RNA with strand displacement and show that if the system is well-mixed (under other simplifying assumptions), there is no selective force acting upon strand preference per se. Next, we construct a spatially-extended individual-based Monte Carlo simulation model using Cellular Automata and investigate the evolution of strand preference in a system of replicators with finite diffusion. Interestingly, the results show that, selective forces "emerge" because of limited diffusion, enabling the evolution of strand preference towards producing single-stranded (+) replicases. Strikingly, however, the direction of the strand preference that evolves turns out to be a complex non-monotonic function of the intensity of diffusion. Finally, we investigate the effect of double-strand complex formation (i.e. a replication intermediate between a double-strand and a replicase) and show that complex formation causes direct selection for preference towards producing single-stranded (-) templates. However, we show that despite complex formation, the evolution of preference towards producing single-stranded (+) replicases is still possible if diffusion is finite and where the decay rate of replicators is sufficiently great.

## Results

### A system with infinite diffusion: a simple mathematical model

In this section, we formulate a simple mathematical model to describe the population dynamics of RNA replicators with strand displacement under the assumption that diffusion is infinite. The model described here serves to illustrate that the emergence of strand preference requires explanation and cannot therefore be treated as a *de facto *feature of polymerisation with strand displacement. Furthermore, it gives us a reference to which more complex models may be compared. A system with a single species is first formulated and is then extended to a system with multiple species.

We consider the following reaction scheme for replication of RNA with strand displacement (see also Fig. 1c):

(1) $$\begin{array}{c} 2\text{P}\overset{k_{SP}}{\underset{}{\to }}\text{P}+\text{D,}\\ \text{P}+\text{M}\overset{k_{SM}}{\underset{}{\to }}\text{P}+\text{D,}\\ \text{P}+\text{D}+\emptyset \overset{k_{DP}}{\underset{}{\to }}\text{P}+\text{D}+\text{M,}\\ \text{P}+\text{D}+\emptyset \overset{k_{DM}}{\underset{}{\to }}\text{P}+\text{D}+\text{P,}\\ \text{P},\text{M},\text{D}\overset{d}{\underset{}{\to }}\emptyset , \end{array}$$

where P denotes a single-stranded (+), which is the replicase; M denotes a single-stranded (-); D denotes a double strand; ∅ represents resources for replication; *k*_*x *_is a reaction rate constant, where *x *denotes templates, namely single-stranded (+) (*x *= *SP*), single-stranded (-) (*SM*), double-stranded (+) (*DP*) and double-stranded (-) (*DM*). The first two reactions represent the production of a double strand through the replication of a single strand as template. We ignore resources involved in this reaction to simplify the models constructed later (this simplification does not affect the general conclusions of the current study; see Authors' response to Reviewer's report 3 for more explanation on this assumption). The next two reactions are the production of a single strand through strand displacement: In the first case, a replicase replicates the (+) strand of a D as template, producing a (-) strand. In the second case, a replicase replicates the (-) strand of a D as template, producing a (+) strand. The last reaction is the decay of molecules, of which rates are assumed to be equal among all molecules for simplicity (we will check this assumption in the CA model; see the last paragraph under the section, "Multi-scale analysis of the model").

Assuming that the system is well-mixed, we can write a simple ordinary differential equation (ODE) that describes the population dynamics of RNA replicators of the above reaction scheme as

(2) $$\begin{array}{c} \dot{P}=-k_{SP}P^{2}+k_{DM}\theta PD-dP,\\ \dot{M}=-k_{SM}PM+k_{DP}\theta PD-dM,\\ \dot{D}=k_{SP}P^{2}+k_{SM}PM-dD, \end{array}$$

where *P*, *M *and *D *denote the concentration of P [single-stranded (+)], M [single-stranded (-)] and D [double strand] respectively; the dots denote time derivative; *θ *takes account of limited multiplication due to a finite supply of resources in general. *θ *can for instance be assumed to take the logistic form for simplicity; i.e. *θ *= 1 - (*P *+ *M *+ *D*), where each concentration is considered to be scaled with respect to the capacity of the system. This assumption is not essential to the results, but makes the Cellular Automata model described in the next section simpler.

Summing both sides for $\dot{P}$, $\dot{M}$ and $\dot{D}$ in Eq. (2), one can obtain

(3) $$\dot{T}=(k_{DM}+k_{DP})\theta PD-dT,$$

where *T *= *P *+ *M *+ *D*. Eq. (3) describes the dynamics of the total concentration.

In order to investigate the dynamics at equilibrium, we assume a quasi-steady state in *D *in Eq. (2):

(4) D* = (*k*_*SP *_*P *+ *k*_*SM *_*M*)*P*/*d*,

where the asterisk denotes a steady state.

Eqs. (3) and (4) enable us to see the general behavior of the model. From Eq. (3), the growth of *T *is proportional to *D*. From Eq. (4), if *k*_*SP *_> *k*_*SM*_, then producing P will increase D* more than producing M.

To put it simply, if one of the single strands is a better template than the other, it is beneficial for multiplication to produce more of that strand. In this case, strand preference is a direct consequence of a preexisting bias in *k*_*SP *_and *k*_*SM*_. Although being rather trivial, the case should not be dismissed when one takes molecular recognition into account (see Discussion). However, for the sake of simplicity, we do not consider molecular recognition processes in our models. For the time being, we assume *k*_*SP *_= *k*_*SM *_(but will later relax this assumption). Then, one can obtain, from Eqs. (3) and (4),

(5) $$\dot{T}=(k_{DM}+k_{DP})\theta P\frac{\chi }{\chi +1}T-dT,$$

where *χ *= (*k*_*SP *_+ *k*_*SM*_)*P/d*. From Eq. (5), one can see that the growth rate of *T *(the first term of the RHS) is proportional to *k*_*DM *_+ *k*_*DP*_. Therefore, unless we assume an intrinsic correlation between the value of *k*_*DM *_+ *k*_*DP *_and the ratio between *k*_*DM *_and *k*_*DP*_, the total growth rate is not directly dependent on the ratio between *k*_*DM *_and *k*_*DP *_itself. However, since the growth rate of *T *is proportional to *P *and to *χ*/(*χ *+ 1) – which increases as *P *increases – the growth rate of *T *does increase if a replicator produces more replicases (P) by biasing the ratio between *k*_*DM *_and *k*_*DP*_. The next question is whether such a bias can evolve through selection.

We next consider a system with multiple species to examine the effect of strand preference on competition (i.e. selection). A system with multiple species is complicated by the inter-species interactions in which both replicases and templates can contribute to the molecular recognition. To fully take account of the evolution of inter-species interactions, we should model a genotype-phenotype-interaction mapping of individual replicators [11] (this would also enable us to take account of a possible bias in *k*_*SP *_and *k*_*SM*_). In this initial study, however, we greatly simplify the system by assuming that replicases do not discriminate between inter-species and intra-species replication, so that the replication rate constants, *k*_*SP*_, *k*_*SM*_, *k*_*DP *_and *k*_*DM*_, are dependent solely on templates. Although this assumption greatly limits the richness of the behavior of replicator dynamics, it enables us to focus on the problem of strand preference (see also Discussion). Under this assumption, we can simply write an ODE model with *n *species as

(6) $$\begin{array}{c} {\dot{P}}_{i}=-k_{SPi}pP_{i}+k_{DMi}\theta pD_{i}-dP_{i},\\ {\dot{M}}_{i}=-k_{SMi}pM_{i}+k_{DPi}\theta pD_{i}-dM_{i},\\ {\dot{D}}_{i}=k_{SPi}pP_{i}+k_{SMi}pM_{i}-dD_{i},\\ \theta =1-\sum_{j=1}^{n}\left(P_{j}+M_{j}+D_{j}\right), \end{array}$$

where $p=\sum_{j=1}^{n}P_{j}$; and subscript *i *and *j *denote species. Realizing that the concentration of P as a replicase (rather than as a template) always appears in Eq. (6) as the summation *p *= ∑_*j *_*P*_*j*_, one can obtain equations similar to Eqs. (3), (4) and (5), respectively, as,

$$\begin{array}{c} {\dot{T}}_{i}=(k_{DMi}+k_{DPi})\theta pD_{i}-dT_{i},\\ D_{i}^{*}=(k_{SPi}P_{i}+k_{SMi}M_{i})p/d, \end{array}$$

and, by assuming *k*_*SPi *_= *k*_*SMi *_again,

(7) $${\dot{T}}_{i}=(k_{DMi}+k_{DPi})\theta p\frac{\chi_{i}}{\chi_{i+1}}T_{i}-dT_{i},$$

where *χ*_*i *_= (*k*_*SPi *_+ *k*_*SMi*_)*p/d*. An important point of this analysis is that the concentration of replicases appears as the sum *p *in Eq. (7). This means that if a replicator *i *produces more replicases by biasing the ratio between *k*_*DM *_and *k*_*DP*_, this increases the growth rate of all replicator species. Hence strand preference does not influence the competition among different species.

In the above analysis, we assumed a quasi-steady state in *D *in order to investigated the dynamics at equilibrium. However, under this assumption, the possibility of non-stationary solutions (such as a limit cycle) is not explored. Hence, we also investigated Eq. (6) numerically without this assumption. We observed no non-stationary solution from the numerical calculation, and the numerical results confirmed the conclusions drawn from the above analysis (data not shown).

In conclusion, under the assumptions that *k*_*SP *_= *k*_*SM *_and that replicases do not discriminate between intra-species and inter-species replication, strand preference per se is not under any selective forces in a well-mixed system, and, therefore, contrary to a prior expectation from the optimization of the growth, its evolution is neutral.

### A system with finite diffusion: a Cellular Automata model

In this section, we will see that if we relax the assumption of infinite diffusion, the above conclusion breaks down: the evolution of strand preference happens selectively. Moreover, we will also see that the direction of the evolution of strand preference is, surprisingly, a *non-monotonic *function of diffusion intensity.

#### The Cellular Automata model

To investigate a system with finite diffusion, we constructed a stochastic Cellular Automata (CA) model. The model is a spatially extended, individual-based, Monte Carlo simulation model. It consists of a two-dimensional square grid and molecules (P or M or D) located on the grid. One square of the grid can contain at most one molecule or be empty (∅). The size of the grid is 300 × 300 squares (spots), unless otherwise stated; the boundary of the grid is toroidal. The temporal dynamics of the model is run by consecutively applying an algorithm simulating the reaction scheme (1) and diffusion. Essential features of the algorithm are that interactions between molecules happen locally – a molecule can interact only with molecules located in eight adjacent squares (Moore neighborhood) – and diffusion happens by swapping of two molecules that are located adjacently on the grid (see Methods for details). The intensity of diffusion is represented by a parameter Δ. Furthermore, each molecule holds four reaction rate constants: *k*_*SP*_, *k*_*SM*_, *k*_*DP *_and *k*_*DM *_as in Reaction (1). Among these, the value of *k*_*SP *_and *k*_*SM *_are fixed (*k*_*SP *_= *k*_*SM*_), while *k*_*DP *_and *k*_*DM *_can have variation, which is introduced by simple perturbation ("mutation"). A mutation can happen upon replication with a certain probability, the mutation rate, denoted by *μ*. In order to separate the effect of the selective force that tends to increase *k*_*DP *_+ *k*_*DM *_from the evolution of the ratio between *k*_*DP *_and *k*_*DM *_[see above and Eq. (7)], we assume that mutations alter the value of *k*_*DM*_/(*k*_*DP *_+ *k*_*DM*_) – this ratio is hereafter denoted by *r *– while keeping *k*_*DP *_+ *k*_*DM *_constant. Such mutations are implemented such that *r *is altered by adding *x *that is uniformly distributed in (-*δ*_*r*_, *δ*_*r*_) [when *r *+ *x *< 0, *r *is set to -*r *- *x*; when *r *+ *x *> 1, *r *is set to 2 -*r *- *x *(i.e. reflecting boundary)].

#### The evolution of template preference in strand displacement

**Figure 2:**
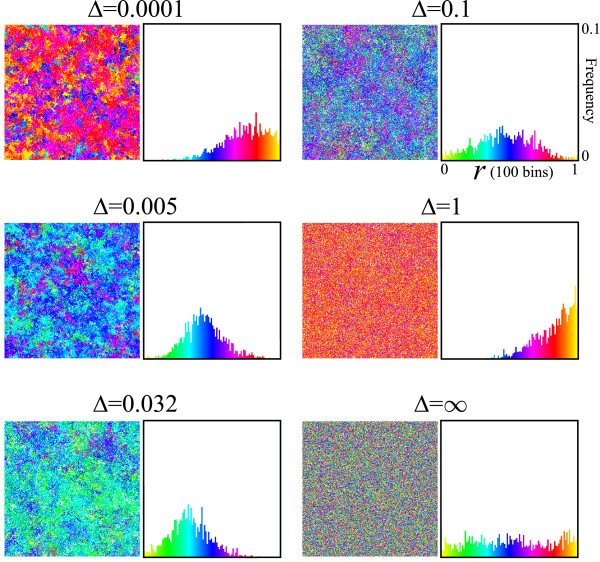
**Snapshots of simulations & population distribution of *r***. The right panels in each pair show a population distribution of *r *at a given time-step of simulations [where *r *= *k*_*DM*_/(*k*_*DM *_+ *k*_*DP*_)]. Population distributions of *r *were observed after the system reached equilibrium. The abscissa is *r *in the range of [0,1] with 100 bins. The coordinate is the frequency of individuals (P or M or D) in the range of [0,0.1] (the sum of frequencies is normalized to 1). The left panels show the spatial distribution of individuals colored by value of *r*. Colors indicate values of *r *at the same time step as that of the population distribution of *r *(right panels). The values of the parameters are as follows: *k*_*SP *_= *k*_*SM *_= *k*_*DP *_+ *k*_*DM *_= 1 (replication rates); *d *= 0.01 (decay rate); *μ *= 0.01 (mutation rate); *δ*_*r *_= 0.1 (mutation step).

Simulations were initialized by filling the grid with P, whose value of *r *is set to 1/2, i.e. no strand preference. Simulations were then run for various diffusion intensity (Δ). Fig. 2 shows snapshots of simulations when the system is at equilibrium. When the value of Δ is extremely small (Δ = 0.0001), the spatial distribution of molecules colored by value of *r *shows a patchy distribution. This is because local reproduction builds up spatial correlation between molecules with the same value of *r*, and diffusion is too weak to disrupt it. The population distribution of *r *is unimodal, and its mean is clearly greater than 1/2 ($\bar{r}$ = 0.74, where the bar denotes the population mean averaged over time). Hence, replicators are preferentially producing single-stranded (+) replicases, optimizing population growth. This is in contrast to the expectation from the ODE model where such optimization is not selected. When, however, the value of Δ is greater (Δ = 0.005), the population mean of *r *becomes almost 1/2 ($\bar{r}$ = 0.48). In this case, replicators do not have strong preference for either strands. When Δ is yet greater (Δ = 0.032), $\bar{r}$ becomes smaller than 1/2. Hence, replicators are preferentially producing single-stranded (-) templates. When Δ is yet greater (Δ = 0.1), $\bar{r}$ increases and again becomes almost 1/2 ($\bar{r}$ = 0.45). However, in contrast to the case of Δ = 0.005 (where $\bar{r}$ is likewise close to 1/2), the population distribution of *r *is much more flat. This flatness indicates that, for Δ = 0.1, selective forces are weaker than those for Δ = 0.005 (however, in both cases, selective forces are balancing around *r *= 1/2). When Δ is even greater, $\bar{r}$ increases to a very high value, and it peaks at as great as 0.84 for Δ = 1. With further increases in Δ, $\bar{r}$ begins to decrease. When Δ = ∞ (see Methods for details), the population distribution of *r *is uniform (thus $\bar{r}$ = 1/2) although it fluctuates. The uniformity of the *r *distribution implies that there is no selective force acting upon *r *(strand preference), which is in perfect concordance to the result of the ODE model.

Next, simulations were run for various decay rates (*d*). During the simulations, the population mean of *r *was measured, and it was averaged over time for each system once it reached equilibrium (denoted by $\bar{r}$).

**Figure 3:**
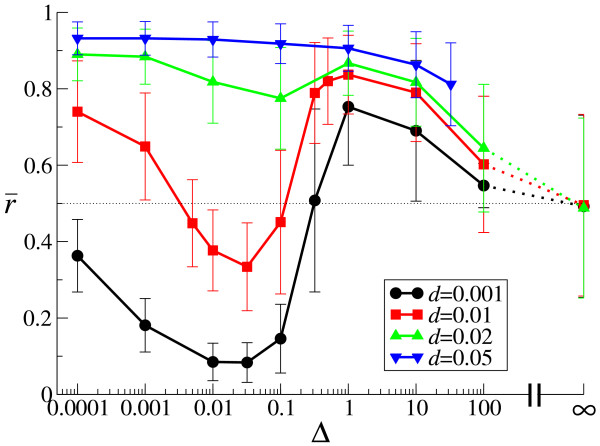
**The evolution of strand preference (*r*) as a function of diffusion intensity (Δ)**. The population mean of *r *($\bar{r}$) is plotted as a function of the diffusion intensity (Δ) for various decay rates (*d*). Error bars show the mean absolute deviation of *r *in a population, which is defined as ADev(*r*) = ∑_*i*_|*r*_*i *_- $\bar{r}$|/*N *where *N *is population size, and *i *denotes an individual. Both $\bar{r}$ and ADev(r) were averaged over time after the system reached equilibrium. The other parameters than Δ and *d *are the same as in Fig. 2. For computational reason, the data for Δ = 100 are obtained from a field of 100 × 100 squares (note that diffusion will be effiectively stronger in a smaller field). The data for great values Δ are not plotted for *d *= 0.05 since the system goes extinct (this is because of the fluctuation in $\bar{r}$, which is due to the system size being finite).

The values of $\bar{r}$ are plotted as a function of Δ for various decay rates (*d*) in Fig. 3. Interestingly, the results show that $\bar{r}$ behaves in a quite complex manner as a function of Δ dependent on the value of *d*. Particularly striking is the non-monotonicity of $\bar{r}$ as a function of Δ when the value of *d *is small (cf. ref. [12]). When the value of *d *is sufficiently great, $\bar{r}$ monotonically decreases to 1/2 as Δ increases. When the value of *d *is intermediate (*d *= 0.02), the behavior of $\bar{r}$ is consistently intermediate too. Finally, for all cases examined, $\bar{r}$ approaches 1/2 as Δ increases to infinity.

In summary, the above results show that if diffusion is not assumed to be infinitely large, strong strand preference can evolve in replicators with strand displacement even if *k*_*SP *_= *k*_*SM*_. The evolved preference can, however, be in either direction (i.e. either (+) strands or (-) strands can be favored) depending on the two crucial parameters, namely the intensity of diffusion (Δ) and the decay rate (*d*).

#### Multi-scale analysis of the model

To understand the above results, we develop a caricature of our full model by separately considering the dynamics at several scales. Firstly, we consider the smallest possible scale, where only two molecules are considered. Secondly, we consider a greater scale which is characterized by a cluster of molecules with the same value of *r *(i.e. a cluster of the same species of replicators). Finally, we consider a yet greater scale characterized by a number of such clusters. Our analysis is based on the combined application of methods developed in several early pivotal studies [13-16] and some extensions thereof.

Our first objective is to calculate the probability that a single-stranded molecule – which can be either plus (P) or minus (M) – is replicated (becoming a double strand, D) in a very small system. Let us consider an isolated sub-system composed of only two squares (spots). Replication – i.e. *P *→ *D *or *M *→ *D *– can happen only if a sub-system contains two P molecules or one P molecule and one M molecule. Such subsystems are denoted by Σ_PP _and Σ_MP _respectively. In such a sub-system, three types of events can happen, namely diffusion, replication and decay. Since these events happen as a Poisson process, the probability that one of these events happen in a given duration of time *τ *can be calculated as 1 - *e*^-(Δ+2*a*+*d*)*τ *^in Σ_PP _and 1 - *e*^-(Δ+*a*+*d*)*τ *^in Σ_MP_, where *a *denotes the rate of replication reaction from single-stranded templates (the production of D), which is proportional to *k*_*SP *_and *k*_*SM *_; and Δ and *d *are respectively the rate of diffusion and decay. Note that a factor of 2 appears in front of *a *for Σ_PP _because there are two possible replication events such that either of the two P molecules can be replicated. Given that some event happens, the conditional probability that the event is replication is calculated as 2*a*/(Δ + 2*a *+ *d*) in Σ_PP _and *a*/(Δ + *a *+ *d*) in Σ_MP_. Therefore, the probability that replication happens in *τ *in Σ_PP _is calculated as

(8) $$(1-e^{-(\Delta +2a+d)\tau })\frac{2a}{\Delta +2a+d},$$

whereas that in Σ_MP _is calculated as

(9) $$(1-e^{-(\Delta +a+d)\tau })\frac{a}{\Delta +a+d}.$$

(See ref. [14] for more details on the calculation.) Since there are two P molecules in Σ_PP_, the probability of replication per P molecule is obtained by dividing Eq. (8) by 2. In Σ_MP_, however, there is only one M molecule, so that the probability of replication per M molecule is the same as Eq. (9). Finally, since *d *has to be smaller than *a *for a surviving system, we can simplify the equations by setting *d *to 0. Consequently, the probability of replication per P in *τ *in Σ_PP _(denoted by $k_{\text{P}|\Sigma_{\text{PP}}}$) is calculated as

(10) $$k_{\text{P}|\Sigma_{\text{PP}}}=(1-e^{-(\Delta +2a)\tau })\frac{a}{\Delta +2a},$$

whereas the probability of replication per M in *τ *in Σ_MP _(denoted by $k_{\text{M}|\Sigma_{\text{MP}}}$) is calculated as

(11) $$k_{\text{M}|\Sigma_{\text{MP}}}=(1-e^{-(\Delta +a)\tau })\frac{a}{\Delta +a}.$$

Now, we set *τ *to the time scale of diffusion such that *τ *= Δ^-1 ^(= *τ*_Δ_) in order to consider the time duration in which two molecules do not diffuse out of the sub-system. Firstly, let us suppose that the time scale of diffusion is much shorter than that of replication; i.e. Δ ≫ *a*. Then,

(12) $$\begin{array}{c} k_{\text{P}|\Sigma_{\text{PP}}}\approx (1-e^{-1})\frac{a}{\Delta },\\ k_{\text{M}|\Sigma_{\text{MP}}}\approx (1-e^{-1})\frac{a}{\Delta }. \end{array}$$

Therefore, the chance of replication is almost equal between P and M. Secondly, let us suppose that the time scale of diffusion (*τ*_Δ _= Δ^-1^) is much longer than that of replication (*τ*_*a *_= *a*^-1^); i.e. Δ ≪ *a*. Then,

(13) $$\begin{array}{c} k_{\text{P}|\Sigma_{\text{PP}}}\approx 1/2,\\ k_{\text{M}|\Sigma_{\text{MP}}}\approx 1. \end{array}$$

In this case, M has a greater chance of replication. Therefore, if two species are competing with each other, it is beneficial to produce more M than P. This advantage of producing M results in selection favouring a decrease in *r*, and can account, in part, for the observation (described in the previous section) that strand preference evolves such that the production of single-stranded (-) templates is favored ($\bar{r}$ < 1/2).

**Figure 4:**
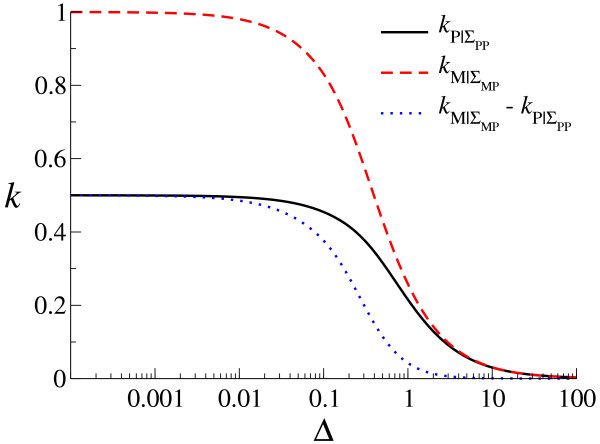
**Advantage of producing (-) strands**. The value of $k_{\text{P}|\Sigma_{\text{PP}}}$ = (1 - *e*^-(Δ+2*a*)*τ*^)*a*/(Δ + 2*a*) (black solid line), that of $k_{\text{M}|\Sigma_{\text{MP}}}$ = (1 - *e*^-(Δ+*a*)*τ*^)*a*/(Δ + *a*) (red dashed line) and the difference thereof (blue dotted line) are plotted as a function of Δ (*τ *is set to Δ^-1^), with *a *= 0.5 [*a *is the rate of replication for single stranded templates – either (+) or (-) strands]. For those plotted values to be applicable to the CA model, *a *should lie between 0.1*k*_*S *_and *k*_*S *_where *k*_*S *_= *k*_*SP *_= *k*_*SM*_. This is because in the CA model the number of neighbors are 8 (rather than 2), and these 8 neighbors are not necesarily all P. Thus, to calculate values corresponding to $k_{\text{P}|\Sigma_{\text{PP}}}$ and $k_{\text{M}|\Sigma_{\text{MP}}}$ in the CA model, one must also factor in the probability that a molecule (P or M) interacts with P given that they are in the neighborhood of the molecule (see Methods for the details of how interactions are implemented in the CA model).

Moreover, as Δ increases from 0 to infinity in Eqs. (10) and (11), a transition is expected to happen from Eq. (13) to Eq. (12) when the order of magnitude of Δ becomes equal to that of *a *(i.e. the rate of second strand synthesis) as shown in Fig. 4 (where *a *is set to 0.5). Indeed, a similar transition is also observed with respect to the evolved value of $\bar{r}$ as shown in Fig. 3 (e.g. for *d *= 0.001), in that $\bar{r}$ suddenly increases when Δ increases from 0.1 to 1 (see also the explanation in Fig. 3).

Next, we consider a whole system, consisting of many sub-systems. The probability of replication per P in a whole system (denoted by *k*_P_) is expressed as

(14) $$k_{\text{P}}=\text{Pr}(\Sigma_{\text{PP}}|\text{P})k_{\text{P}|\Sigma_{\text{PP}}},$$

whereas the probability of replication per M in a whole system (denoted by *k*_M_) is expressed as

(15) $$k_{\text{M}}=\text{Pr}(\Sigma_{\text{MP}}|\text{M})k_{\text{M}|\Sigma_{\text{MP}}},$$

where Pr(Σ_PP_|P) denotes the probability that a P molecule co-occurs with another P molecule in a sub-system; Pr(Σ_MP_|M) denotes the probability that a M molecule co-occurs with a P molecule in a sub-system. The probabilities Pr(Σ_PP_|P) and Pr(Σ_MP_|M) represent the degree of spatial correlation between molecules [15]. Although it is almost impossible to calculate these quantities because of inhomogeneities at multiple scales [16], we can still obtain some intuitive ideas about our system from Eqs. (14) and (15). Let us suppose that there are two species competing with each other, where species 1 produces only P, whereas species 2 produces only M (i.e. *r*_1 _= 1 and *r*_2 _= 0, where the subscripts denote species). Where Δ = ∞, molecules are randomly distributed among the sub-systems. Hence, Pr(Σ_PP_|P_1_) = Pr(Σ_MP_|M_2_) = *P*, where *P *is the density of P in the whole system (i.e. the total number of P divided by the total number of squares). Therefore, the probability of interacting with replicases (P) is "fair" between two species. If, however, Δ = 0, this situation disappears: the molecules of species 1 (P producers) will interact with replicases more frequently than those of species 2 (M producers), since there will be strong positive spatial correlation within the same species because of local reproduction. Therefore, if diffusion is finite, there is an advantage in preferentially producing P.

In summary, when diffusion is finite, there is an advantage of producing M because of persistent interactions, and there is an advantage of producing P because of spatial correlation. Both of these two opposing advantages diminish as the intensity of diffusion (Δ) increases. Therefore, the crucial question is how each of these two advantages does so relative to each other.

**Figure 5:**
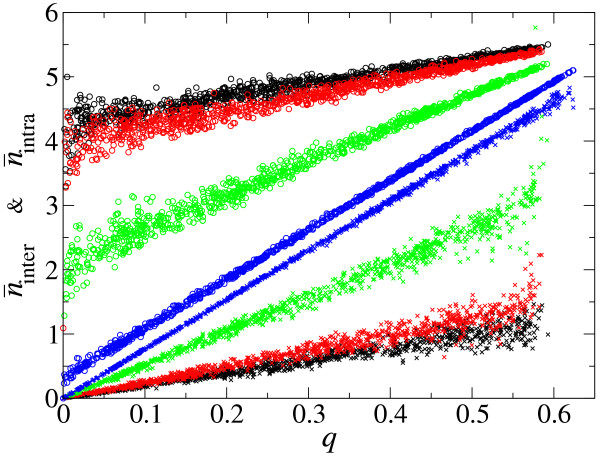
**The degree of spatial correlation within same species and between different species as a function of the density of observed species**. ${\bar{n}}_{\text{intra}}$ (circles) is the average number of molecules of the same species that a given molecule "meets" in a single time-step (i.e. that are in its neighborhood) (see main text for details). The abscissa (*q*) is the density of the same species, which is calculated as the number of individuals of that species divided by the total number of squares on the grid. ${\bar{n}}_{\text{inter}}$ (crosses) is the average number of molecules of the opposite species that a given molecule meets in a single time-step. The abscissa (*q*) is the density of the opposite species, which is calculated in a manner similar to the case of ${\bar{n}}_{\text{intra}}$. The two species are identical with respect to the parameters, and both have *r *= 1/2. Colors represent diffusion intensity (Δ): Δ = 0.001 (black); Δ = 0.01 (red); Δ = 0.1 (green); Δ = 1 (blue). The rate of decay (*d*) is 0.05. Mutation is disabled (*μ *= 0). The other parameters are the same as in Fig. 2. A tentative explanation for why ${\bar{n}}_{\text{intra}}$ is a linear function of *q *can be given as follows. Because of the local reproduction, when a species exists, it always exists on the grid as aggregates. Therefore, an individual always "meets" some number of individuals of the same species no matter what the density of the species in a whole system is (*α *> 0). When an aggregate "meets" with other aggregates, then the individuals will see more individuals of the same species, which increases ${\bar{n}}_{\text{intra}}$. Given that the aggregates are randomly distributed on the grid, the chance of an aggregate meeting with another aggregate is proportional to the number of aggregates in the system, which is proportional to the total density of the species (*q*). Therefore, ${\bar{n}}_{\text{intra}}$ is a linear function of *q*. Because of the symmetry, ${\bar{n}}_{\text{inter}}$ is also a linear function with the same slope as ${\bar{n}}_{\text{intra}}$ (but the intercept must obviously be 0 for ${\bar{n}}_{\text{inter}}$.

The advantage of producing M has already been examined as a function of Δ (Fig. 4), so we now measure the advantage of producing P as a function of Δ. For this sake, the following simulations were set up. The CA model was initialized with replicators with identical parameters; in particular, *r *was set to 1/2. Each molecule was labeled as either 1 or 2, and these labels were copied when molecules were replicated. Hence, the labels represent "species". Simulations were then run as before except that mutation is prohibited. Then, we measure, for each molecule at a given time-step, how many molecules of the same species (denoted by *n*_intra_) and how many molecules of the other species (denoted by *n*_inter_) are in the neighborhood of the molecule (see Methods for details). *n*_intra _represents the number of molecules of the same species one molecule "meets" in one time-step, whereas *n*_inter _represents the number of molecules of the other species one molecule meets in one time-step. The population mean of those quantities (denoted by ${\bar{n}}_{\text{intra}}$ and ${\bar{n}}_{\text{inter}}$) are periodically calculated in a sufficiently long span of time-steps to ensure the independence between data points. The results of measurements are shown in Fig. 5, where ${\bar{n}}_{\text{intra}}$ is plotted as a function of the density of the same species when ${\bar{n}}_{\text{intra}}$ is measured, whereas ${\bar{n}}_{\text{inter}}$ is plotted as a function of the density of the other species when ${\bar{n}}_{\text{intra}}$ is measured (note that, in both cases, the abscissa is denoted by *q*). Interestingly, it turns out that ${\bar{n}}_{\text{intra}}$ and ${\bar{n}}_{\text{inter}}$ are a linear function of *q *for the intermediate values of *q*, and the two functions have almost the same slope (see legend to Fig. 5 for an explanation). Therefore, for intermediate values of *q*, we can write

(16) $$\begin{array}{c} {\bar{n}}_{\text{intra}}=\alpha +\beta q,\\ {\bar{n}}_{\text{inter}}=\beta q, \end{array}$$

where *α *and *β *can be obtained by a linear regression on the data from intermediate values of *q*.

Let us consider the meaning of *α *and *β*. If *α *> 0, then it means that molecules always "see" an appreciable amount of its own species however small its density is in the whole system (note that the range of *q *for which ${\bar{n}}_{\text{intra}}$ is linear with respect to *q *is expected to expand as the size of the system increases). Therefore, we can say that *α *represents the critical degree of aggregation. *α *can thus be used as a measure of the advantage of producing P, whereas *β *represents the sensitivity of ${\bar{n}}_{\text{intra}}$ and ${\bar{n}}_{\text{inter}}$ when the value of *q *changes.

**Figure 6:**
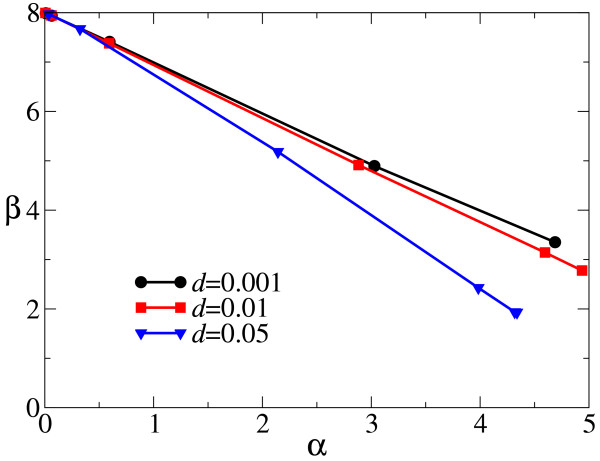
**Relationship between the critical degree of aggregation (*α*) and the sensitivity of correlation to the density of observed replicators (*β*)**. *α *and *β *are calculated as, respectively, the intercept and slope of the linear regression to ${\bar{n}}_{\text{intra}}$ for intermediate values of *q *for which ${\bar{n}}_{\text{intra}}$ is approximately linear to *q *(see Fig. 5). Values of *d *are as shown in the plot. For the same value of *d*, several data points are plotted for different values of Δ (from right to left, Δ = 0.0001, 0.001, 0.01, 0.1, 1, 10; points are almost on top of each other for Δ = 1 and 10). The other parameters are the same as in Fig. 5. Note that *α *can also be calculated from ${\bar{n}}_{\text{inter}}$ since ${\bar{n}}_{\text{inter}}$ has almost the same slope as that of ${\bar{n}}_{\text{intra}}$, as shown in Fig. 5. Also note that when *α *≈ 0 (i.e. when Δ ≫ 1), *β *becomes almost 8 because the current CA model uses Moore (8) neighbors.

To reveal the relationship between *α *and *β*, the two are measured for various values of Δ and *d*. As seen from Fig. 6, it turns out that for a given value of *d*, *α *and *β *are lineally related for different values of Δ.

Phenomenologically, this can be seen from Fig. 5, in that, for different values of Δ, ${\bar{n}}_{\text{intra}}$(*q*) intersects at about the same point, (*q*^*c*^, ${\bar{n}}_{\text{intra}}^{c}$), where *q*^*c *^is the density of a species when there is only this species in the system, and ${\bar{n}}_{\text{intra}}^{c}$ is the value of ${\bar{n}}_{\text{intra}}$ when *q *= *q*^*c*^. Since *q*^*c *^is more or less invariant as a function of Δ – which is not totally inconceivable in a contact-process-like system [16] – ${\bar{n}}_{\text{intra}}^{c}$ is also more or less invariant with respect to Δ. Hence, there is an almost linear relationship between *α *and *β *for various values of Δ: *α *+ *βq*^*c *^= ${\bar{n}}_{\text{intra}}^{c}$ (Fig. 6). Because of this linear relationship, the degree to which individuals of the same species are aggregated for a given decay rate can be described by one value, the critical degree of aggregation *α*, which is also a measure of the advantage of producing single-stranded (+).

**Figure 7:**
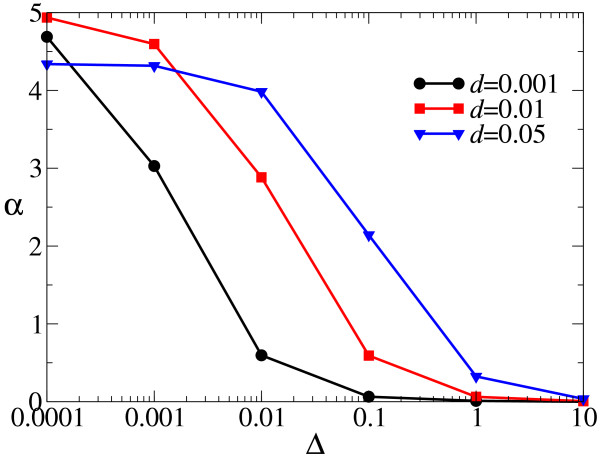
**Advantage of producing (+) strands: the critical degree of aggregation (*α*) as a function of the diffusion intensity (Δ)**. *α *is plotted as a function of Δ by using the data of Fig. 6. This figure shows that the characteristic value of *α *for which a decreases depends on the value of *d*.

**Figure 8:**
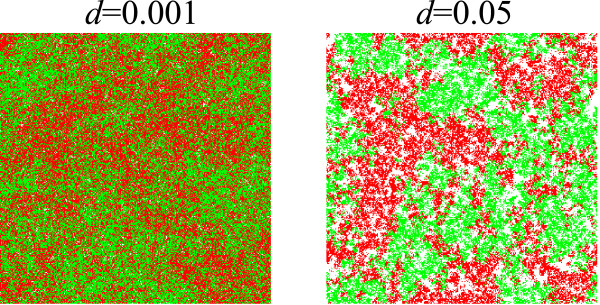
**The effect of decay on the spatial distribution of replicators**. Snapshots of simulations show a spatial distribution of replicators for different decay rates (*d*). The two colors represent two different "species", which have identical parameters (see "Multi-scale analysis of the model" under Results for more details). The intensity of diffusion (Δ) is 0.01. The other parameters are the same as in Fig. 5. The snapshots are taken when the frequency of two species is almost fifty-fifty. This figure shows that a greater decay rate (shorter longevity) of replicators leads to a greater spatial correlation within the same species.

Next, the value of *α *was measured for various values of *d*, and the results are plotted as a function of Δ in Fig. 7. Interestingly, the characteristic value of Δ for which *α *critically diminishes (i.e. the point of inflexion) increases as *d *increases. We can intuitively understand this from the fact that the mean square displacement of a molecule in its average life time is proportional to $\sqrt{\Delta /d}$. That is, as *d *increases, molecules travel less. Since replication happens locally, if molecules travel a shorter distance prior to decay, mixing of molecules effectively occurs less. The effect of increasing *d *is also clearly seen from the spatial distribution of two species for different decay rates as shown in Fig. 8. Therefore, as *d *increases, the characteristic value of Δ (for which *α *diminishes) increases.

To summarize the essence of the above results, the advantage of producing M diminishes as the diffusion intensity (Δ) increases, and the characteristic point for which this advantage critically diminishes is when Δ and the effective rate of replication [*a *in Eqs. (8) and (9)] are of the same order of magnitude (Fig. 4). Here, the characteristic point is almost independent of the decay rate (*d*) because *d *appears in Eqs. (8) and (9) through addition to Δ and *a*, where *a *is greater than *d *for surviving systems. The advantage of producing P also diminishes as Δ increases; however, the characteristic value of Δ for which the advantage of producing P diminishes now depends more strongly on the decay rate (*d*), because it depends on the mean square displacement of – i.e. distance traveled by – a molecule in its average life time, which is proportional to $\sqrt{\Delta /d}$.

Let us now explain the result of the full system shown in Fig. 3. When the value of *d *is small (*d *≤ 0.02), the characteristic value of Δ for which the advantage of producing P diminishes is smaller than that for which the advantage of producing M diminishes (compare Figs. 4 and 7). Therefore, when Δ is small, there is a range of Δ for which the evolved value of $\bar{r}$ decreases as Δ increases. When, however, Δ is around the same order of magnitude as the replication rate, the advantage of producing M critically diminishes as Δ increases (Fig. 4). This causes $\bar{r}$ to increase with increases in Δ. It might be expected from our caricature that when the value of Δ exceeds the replication rate by an order of magnitude, $\bar{r}$ should become 1/2 because both the advantage of producing P and that of producing M largely diminish. However, the full model shows more than this: $\bar{r}$ actually becomes greater than 1/2 and peaks around Δ = 1 (Fig. 3). Thus, the advantage of producing P is apparently still stronger compared to that of producing M in this region of Δ, even though the value of *α *is quite small (see, e.g. *d *= 0.001 and Δ = 1 in Fig. 7). It should of course be noted that the value of *α *and the difference between the value of Eqs (10) and (11) (Fig. 4) cannot be directly compared with each other in regard to the actual selective forces. Moreover, it is worth noting that the advantage of producing P affects both reaction P → D and reaction D → P, whereas the advantage of producing M only affects reaction M → D. With further increases in Δ, the system starts to resemble a well-mixed system, and $\bar{r}$ approaches 1/2. When the value of *d *is great (e.g. *d *= 0.05), the value of Δ for which the advantage of producing P critically diminishes is about the same as that for the advantage of producing M. Thus, the evolved value of $\bar{r}$ monotonically decreases to 1/2 as Δ increases.

In essence, the reason why the evolved value of $\bar{r}$ can behave non-monotonically with respect to the intensity of diffusion is that the advantage of producing single-stranded (+) and that of producing single-stranded (-) can diminish at different diffusion intensities (Δ) because of their different sensitivity to the decay rate (*d*).

We also investigated the case where the assumption that all molecules decay with an equal rate is relaxed. In reality, the decay rate of D would be smaller than that of P and M (Reviewer's report 3; ref. [17]). To check whether our general conclusions remain valid when this point is taken into consideration, we ran simulations in which the decay rate of D is reduced by a factor of ten relative to that of P and M. The results showed that although there are quantitative differences, the qualitative behavior of $\bar{r}$ as a function of Δ remains the same and is compatible with the interpretation outlined above (see Fig. 11 of Additional file 1 and Author's response to Reviewer's report 3, for more details).

### The effect of biases in the replication rates of single strands (a case where *k*_*SP *_≠ *k_*SM*_*)

In this section, we extend the investigation of strand preference evolution to the case where we relax the assumption that the replication rates of single-stranded templates (i.e. production of double strands) are equal whether a single-stranded (+) or a single-stranded (-) is a template – i.e. we here investigate the case where *k*_*SP *_≠ *k*_*SM*_. We first investigate the case where *k*_*SP *_and *k*_*SM *_take some constant values before turning to a system wherein *k*_*SP *_and *k*_*SM *_evolve. In each of these cases, the inequality between *k*_*SP *_and *k*_*SM *_causes some additional selective force, which can be identified initially by investigating a well-mixed system. We then investigate the effects of imposing finite diffusion, in light of the effect this had for the case where *k*_*SP *_= *k*_*SM*_. Results were obtained from the CA model where almost all the conditions are as before, except that the size of the system is now smaller (100 × 100 squares) – this does not qualitatively affect the results.

If we set *k*_*SP *_and *k*_*SM *_to some constant values with infinite diffusion (Δ = ∞), we find that if *k*_*SP *_is greater than *k*_*SM *_by just a few percent, $\bar{r}$ evolves to a value much greater than 1/2. If, however, *k*_*SM *_> *k*_*SP *_(again, by no more than a few percent), evolution decreases $\bar{r}$ until the whole system goes extinct (data not shown). These results show that a preexisting bias in *k*_*SP *_and *k*_*SM *_introduces an explicit selective force for a bias in *k*_*DP *_and *k*_*DM *_as is consistent with the expectation from the ODE model [see the explanation of Eqs. (3) and (4)]. If diffusion is finite, the results are simply determined by the relative superposition of this selective force from *k*_*SP *_≠ *k*_*SM *_and the selective forces resulting from diffusion being finite explained in the earlier sections. In particular, it is worth reporting that sufficiently small diffusion prevents the extinction of a whole system even if *k*_*SM *_is several times greater than *k*_*SP *_(e.g. if *k*_*SM *_= 1.7, *k*_*SP *_= 0.3, *d *= 0.001 and Δ = 0.01, then $\bar{r}$ ≈ 0.07).

We next investigate cases where *k*_*SP *_and *k*_*SM *_can evolve. Since we do not explicitly consider molecular recognition processes in the current model, we choose to investigate the system under two extreme assumptions in order to capture the widest range of possible behaviors. The first of the two assumptions is that there is complete correlation between strand preference in single strands and that in double strands; i.e. 0.5*k*_*SP *_= *k*_*DP *_and 0.5*k*_*SM *_= *k*_*DM *_(where *k*_*DP *_and *k*_*DM *_mutate while satisfying *k*_*DP *_+ *k*_*DM *_= 1 as before). If Δ = ∞, $\bar{r}$ evolves towards 1/2 (data not shown). This is because if a replicator has *k*_*DM *_> *k*_*DP *_(i.e. producing more P from D), then it also has *k*_*SM *_> *k*_*SP *_(i.e. producing less D from P), which then decreases the overall replication rate. The same is true (i.e. $\bar{r}$ evolves towards 1/2) if *k*_*DP *_> *k*_*DM*_. Hence, there is an explicit selective force towards no strand preference (*r *= 1/2). If, however, diffusion is finite, the results are again determined by the superposition of this selective force and the selective forces resulting from diffusion being finite. It turns out that the force of selection towards *r *= 1/2 is so strong that the deviation of $\bar{r}$ from 1/2 is small (e.g. if *d *= 0.001 and Δ = 0.01, then $\bar{r}$ ≈ 0.30; if *d *= 0.05 and Δ = 1, then $\bar{r}$ ≈ 0.62).

The second assumption we investigate is that there is no intrinsic correlation between the strand preference in single strands and that in double strands. Here we assume that 0.5(*k*_*SP *_+ *k*_*SM*_) is constant and equals *k*_*DP *_+ *k*_*DM *_(= 1). If Δ = ∞, evolution maximizes either *k*_*SP *_and *k*_*DM *_or *k*_*SM *_and *k*_*DP *_(in the latter case the whole system eventually goes extinct) (data not shown). In other words, anti-correlation evolves between the strand preference in single strands and that in double strands; furthermore, this evolution is bistable. Which attractor the system evolves to depends on the initial value of *r*: If *r *is initially set to greater than 1/2, maximizing *k*_*SP *_and *k*_*DM *_is far more likely (i.e. increasing *r*). Similarly, where *r *is initially smaller than 1/2, maximizing *k*_*SM *_and *k*_*DP *_is more likely. These results can simply be understood from the fact that there is an evolutionary positive feedback between increasing *k*_*SP *_and increasing *k*_*DM*_, and between increasing *k*_*SM *_and increasing *k*_*DP *_(see Fig. 1c). Hence, selection drives *r *to diverge from 1/2.

If Δ is finite, the results can again be explained by the relative strength of this diverging selective force and the selective force stemming from diffusion being finite. If Δ is large but finite (Δ ≥ 1), the results are similar to the case of Δ = ∞, but the range of initial values of *r *for which the whole system goes extinct becomes narrower, because selection due to finite diffusion favors *r *> 1/2 in this parameter region (see Δ = 1 in Fig. 3). When Δ is smaller (Δ <1), various outcomes are possible. The most important difference caused by diffusion being finite is that the extinction of a whole system is prevented by the local extinction of the populations that completely maximize *k*_*SM *_and *k*_*DP *_(i.e. *r *≈ 0); e.g. if Δ = 0.01 and *d *= 0.001, then $\bar{r}$ ≈ 0.06, and the system survives.

**Figure 9:**
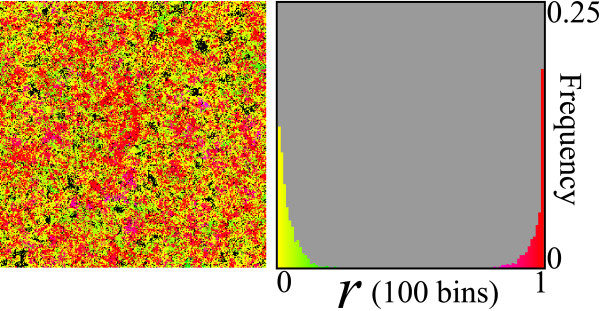
**Speciation: emergence of replicase transcribers (*r *≈ 1) and parasitic genome copiers (*r *≈ 0)**. This figure shows the results of a simulation where *k*_*SM *_and *k*_*SP *_can also evolve the correlation with *k*_*DM *_and *k*_*DP *_is not presumed, but it may evolve. The right panel shows a population distribution of *r*. The abscissa is *r *in the range of [0,1] with 100 bins. The coordinate is the frequency of individuals in the range of [0,0.25]. A bimodal distribution indicates a speciation-like phenomenon [the population distribution of *k*_*SP*_/(*k*_*SP *_+ *k*_*SM*_) also shows a similar bimodal distribution; data not shown]. The left panel shows the spatial distribution of individuals colored by value of *r*. The color coding is the same as in the right panel (note that the color coding is different from that of Fig. 2). For the sake of comparison, the size of the grid is set to 300 × 300 as in Fig. 2. The parameters are as follows: 0.5(*k*_*SM *_+ *k*_*SP*_) = (*k*_*DM *_+ *k*_*DP*_) = 1; Δ = 0.01; *d *= 0.01; *μ *= 0.01; *δ*_*r *_= 0.1. The mutation of *k*_*SM *_and *k*_*SP *_is implemented in the same way as that of *k*_*DM *_and *k*_*DP*_. [Additionaly, we observed the speciation-like phenomenon also in the following parameter conditions: *d *= 0.001 and 0.1 ≤ Δ ≤ 0.32; *d *= 0.01 and 0.001 ≤ Δ ≤ 0.1; *d *= 0.02 and Δ = 0.1.].

A particularly interesting result observed with finite diffusion (under certain conditions) is the emergence of a bimodal distribution of *r *(Fig. 9), from which an analogy can be drawn with speciation [18]. Although the precise conditions for this speciation-like phenomenon has not been completely elucidated, it seems that a bimodal distribution can evolve when the advantage of producing P and that of producing M – which result from diffusion being finite – are sufficiently and more or less equally strong (see the legend of Fig. 9 for the parameter conditions). These opposing advantages and the diverging selection, which was explained above, together make the speciation-like phenomenon possible. Firstly, the opposing advantages enable the stable coexistence of replicators with extreme values of *r*, namely *r *≈ 1 and *r *≈ 0. An individual of *r *≈ 0 can invade the region of populations of *r *≈ 1 because of the former's advantage in producing M. However, populations of *r *≈ 0 cannot survive independently (as they lack replicases), so that, locally, they go extinct (see the left panel of Fig. 9). This enables the continuous local recolonization of individuals of *r *≈ 1. Secondly, under diverging selection, those replicators with no strand preference (*r *≈ 1/2) are disadvantaged relative to those with extreme strand preference (this is not the case under the assumption that *k*_*SP *_= *k*_*SM *_= const., where this speciation is thus prohibited). Consequently, these two effects together cause the observed speciation-like phenomenon.

Besides the above results, we mention two additional results from the simulations with finite diffusion. Firstly, the anti-correlation between the strand preference in single strands and that in double strands generated by evolution under finite diffusion turns out to be non-linear (see Fig. 12 of Additional file 1 for details). Secondly, if the selection stemming from diffusion being finite is too strong compared to the diverging selection mentioned above, the bistability of evolution is lost (e.g. for *d *= 0.001, 0.02 and Δ = 0.0001, the bistability was not observed).

In summary, a bias in the replication of single-stranded templates (i.e. a strand preference in double strand production from single-stranded templates) leads to selection generating anti-correlated strand preference in strand displacement of double strands. The final outcome of evolution under finite diffusion can be understood by applying the results of the preceding sections in addition to the consideration of this selective force for the anti-correlation.

### The effect of complex formation

We now examine the effect of complex formation between replicases and templates. The effect of complex formation on the stability of RNA-like replicator systems was investigated previously [12,19], where it was shown that complex formation generally disadvantages replicases [19], although this does not necessarily destabilize a whole system [12,19]. A simple explanation of this is that replicases must spend some finite amount of time replicating other molecules, during which the replicases themselves are not replicated, whereas those that do not replicate other molecules (parasites) can spend more time being replicated. In view of that result, it is of interest to study how complex formation influences the evolution of strand preference in the current replicator system.

The reaction scheme considered here is

(17) $$\begin{array}{c} 2\text{P}\overset{k_{SP}}{\underset{}{\to }}\text{P}+\text{D},\\ \text{P}+\text{M}\overset{k_{SM}}{\underset{}{\to }}\text{P}+\text{D},\\ \text{P}+\text{D}+\emptyset \overset{k_{DP}}{\underset{b}{⇌}}{\text{ C}}_{\text{P}}\overset{\kappa }{\underset{}{\to }}\text{P}+\text{D}+\text{M},\\ \text{P}+\text{D}+\emptyset \overset{k_{DM}}{\underset{b}{⇌}}{\text{ C}}_{\text{M}}\overset{\kappa }{\underset{}{\to }}\text{P}+\text{D}+\text{P},\\ \text{P},\text{M},\text{D},{\text{C}}_{\text{P}},{\text{C}}_{\text{M}}\overset{d}{\underset{}{\to }}\emptyset , \end{array}$$

where C_P _and C_M _denote a complex molecule formed between P and D. In C_P_, the replicase (P) is polymerizing RNA using the (+) strand of D as a template, whereas in C_M_, the replicase (P) is polymerizing RNA using the (-) strand of D as a template. For simplicity, complexes between replicase and either of the possible single-stranded templates are not considered. An ODE model and a CA model were constructed by extending the models described above (see Methods for details). As before, it is assumed that *k*_*SP *_= *k*_*SM*_, that *k*_*DP *_+ *k*_*DM *_is constant, and that replicases do not discriminate templates (i.e. *k*_*SP*_, *k*_*SM*_, *k*_*DP *_and *k*_*DM *_are solely dependent on templates).

We numerically investigated the competition between two replicator species with different values of *r *in the ODE model. For all cases examined, it is always the case that the species with a smaller value of *r *survives, while the other goes extinct over a very long time scale relative to the average lifetime of replicators (*d*^-1^) (data not shown). Where a replicator species has a value of *r *too small to survive by itself, the whole system goes extinct. In conclusion, in a well-mixed system, if complex formation is taken into account, it is always beneficial to preferentially produce (-) strands. This result is in concordance with a previous study [19], which considered complex formation in simpler RNA-like replicator systems.

We next investigated the evolution of *r *under finite diffusion in a CA model. Simulations were run for the same initial conditions (except that, in some simulations, *r *was initially set to a value greater than 1/2 in order to prevent extinction at the initial phase of the simulation).

**Figure 10:**
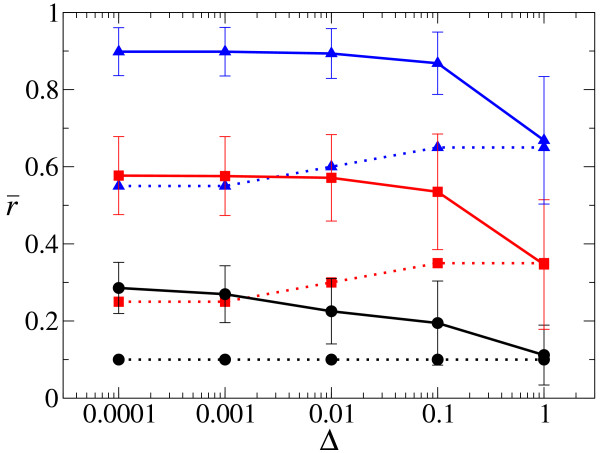
**The evolution of strand preference (*r*) as a function of the diffusion intensity (Δ) in the system with complex formation**. Solid lines represent the evolved value of $\bar{r}$ as a function of the diffusion intensity (Δ) for various decay rates (*d*). Dashed lines represent the minimum value of *r *necessary to ensure system survival (*r*_min_). Colors (and simbols) represent the value of *d*: *d *= 0.0025 (black circles); *d *= 0.0125 (red squares); *d *= 0.025 (blue triangles). The other parameters are as follows: *k*_*SP *_= *k*_*SM *_= *k*_*DP *_+ *k*_*DM *_= 1; *b *= 1; *κ *= 1; *μ *= 0.01; *δ*_*r *_= 0.1.

The results show the following (Fig. 10). When the value of Δ is sufficiently great, the system goes extinct, as in the ODE model. This is because the evolved value of $\bar{r}$ decreases as Δ increases, and at a certain point $\bar{r}$ becomes smaller than the minimum value of *r *(*r*_min_) necessary to ensure system survival (note that *r*_min _increases as Δ increases; Fig. 10 dashed lines). When, however, the value of Δ is sufficiently small, the system can survive. This is because the advantage of producing P due to local aggregation keeps the evolved value of $\bar{r}$ greater than *r*_min_. Moreover, the non-monotonic behavior of $\bar{r}$ with respect to Δ is lost, which can be explained as follows. In the current system, the advantage of producing M does not diminish at Δ = *a *(i.e. when the diffusion intensity is equal to the rate of replication for double strand formation from a single-stranded template), because the advantage of producing M due to complex formation is not affected by the value of Δ. The advantage of producing P, however, does diminish as Δ increases for the same reason as we saw in the earlier sections (the degree of local aggregation diminishes as Δ increases). Therefore, the behavior of $\bar{r}$ is monotonic with respect to Δ.

Secondly, the value of $\bar{r}$ heavily depends on the value of *d*. This is understood in light of the earlier sections, because the critical degree of aggregation (*α*) increases as *d *increases. The results shown in Fig. 10 indicate that $\bar{r}$ can exceed 1/2 if *d *is sufficiently large (e.g. *d *= 0.005). This is not necessarily the case when the parameter setting is different. For instance, when the rate of dissociation of complexes (*b*) [see reaction scheme (17)] is set to 0, the evolved value of $\bar{r}$ is always below 1/2 (data not shown). In this case, the equilibrium of reaction P + *D *⇌ C is completely shifted to the right side, so that the effect of complex formation is extremely strong [19]. However, when such a system is well-mixed, it goes extinct due to complex formation. Hence, the effect of aggregation (non-zero *α*) is still effective in keeping the system alive in this extreme parameter choice.

## Discussion

The current study investigated the evolution of strand preference in simple RNA-based replicators with strand displacement. It was shown that the evolution of strand preference can and does occur, and that its direction depends on four crucial parameters. The first of those is the relative time-scale of diffusion and replication [Eqs. (10) and (11)], which is related to the relative impact of global and local dynamics. When the time-scale of replication (namely, double strand production from single-stranded templates: *k*_*SP *_and *k*_*SM *_; see Reaction 1) is much faster than that of diffusion, a template has an advantage because it ensures its own replication by not replicating the replicases, whereas a replicase reduces the chance of its own replication because it converts the other replicases into a double-stranded form. As the rate of diffusion increases, this advantage of templates disappears, because in a well-mixed system every replicator interacts with every other replicator, so that whether or not a given replicator is engaged in replicating other molecules does not affect the probability of its own replication. The second crucial parameter is the relative time-scale of diffusion and decay. When the rate of diffusion is smaller than that of decay, the distance traveled by a replicator in its lifetime will be short. Consequently, local reproduction will cause stronger positive correlation in the spatial distribution of replicators of the same descent. This positive correlation makes it advantageous to produce replicases rather than templates, since the replicases are more likely to replicate their own kind. Interestingly, this advantage of producing replicases and the advantage of producing templates mentioned above are related via the intensity of diffusion, in that both of these opposing advantages diminish as the intensity of diffusion increases. The diminishment of these two advantages is, however, differentially sensitive to the diffusion intensity, because the two advantages have different dependency on the decay rate of replicators. The upshot of all this is that the evolution of strand preference can be a non-monotonic function of the intensity of diffusion. The third crucial parameter is the (intrinsic or possible) bias in the replication of single-stranded templates (i.e. a bias in double strand production from single-stranded templates; *k*_*SP *_≠ *k*_*SM*_). The essence of the results here is quite straightforward: if there is a bias in the replication of single-stranded templates, this tends to cause anti-correlated strand preference in strand displacement of double strands (*k*_*SP *_<*k*_*SM *_causes *k*_*DP *_> *k*_*DM*_, whereas *k*_*SP *_> *k*_*SM *_causes *k*_*DP *_> *k*_*DM*_), which optimizes the total process of self-multiplication. The last of the crucial parameters is complex formation. Complex formation gives an implicit advantage to those replicators that preferentially produce templates [19]. This effect makes a whole system collapse if the system is well-mixed. However, extinction can be prevented if diffusion is finite because of spatial correlation. In fact, there are a range of parameters for which strand displacement can evolve preference for replicase production.

The evolution of strand preference has implications for the origin of transcription in an RNA world. During the "life cycle" of the RNA replicators investigated here, RNA molecules go through both single-stranded and double-stranded form. Single-stranded (+) strands are the catalyst which generates replicators, whereas double-stranded RNA can be considered the genome of the replicators. Hence, the production of replicases from a double-strand (via strand displacement) can be seen as the expression of the replicase "gene" in the genome of the replicator. There is thus a distinction between genotype and phenotype even in a simple replicator system. In the event that strand displacement exhibits strong preference towards the (-) strand as template (thus preferentially producing replicases), such a system resembles modern transcription. The current study demonstrates that such transcription-like pattern in strand preference can indeed evolve in a simple RNA-based replicator system under the conditions mentioned above. Interestingly, we also see the emergence of a parasitic genome replication strategy where no replicase is ever produced. In the absence of replicases, this is inviable, but under some conditions (see Fig. 9), finite diffusion enables the coexistence of such replicators with replicase "transcribers" (replicase producers), where the parasitic strategy makes use of replicases produced by the transcriber strategy. Note, however, that one should not equate the distinction of replicase transcribers and parasitic genome copiers to the distinction of transcription and (genome) replication in modern genetic systems. Despite their superficial resemblance, the two kinds of distinction are crucially different, for transcription and replication in modern genetic systems are mutually exclusive (transcription does not multiply the genome; replication does not produce mRNA), whereas the two extreme strategies in the current system are both pertaining to the genome replication process. Nevertheless, it is surprising to see the emergence and coexistence of the two strategies based on extreme strand preference among competing replicators.

We now turn to a brief discussion of the simplifying assumptions made in the current model. First, we assumed that replicases are general so as not to discriminate templates of different replicators. While this assumption is useful for investigating the evolution of strand preference per se, allowing the evolution of replicase-template recognition would make the behaviors of a replicator system much more complex and interesting [11]. This point is particularly important in relation to the emergence of the parasitic genome copier, because parasitic agents can enhance the ecological diversity of replicator systems if the evolution of replicase-template recognition is allowed (op. cit.). Second, we assumed *k*_*DP *_+ *k*_*DM *_to be independent of *k*_*DM*_/(*k*_*DM *_+ *k*_*DP*_), which is a necessary assumption in order to separate the effect of strand preference from selection to increase *k*_*DP *_+ *k*_*DM*_. This assumption means that there is direct competition between *k*_*DP *_and *k*_*DM*_. Such competition could for instance be manifested as a series of conformational changes between interacting replicase and template until an intermolecular conformation that enables polymerization initiation is reached. Third, we did not here consider the strand preference that may originate from the intrinsic nature of molecular recognition. Such an intrinsic bias should not be dismissed if one is to take explicit account of molecular recognition processes. For example, let us suppose that the recognition between replicases and templates happens through interactions between two types of motifs, namely one for recognizing and the other for being recognized [19]. Then, for a replicase to be also a template, it must carry both types of motif. Consequently, there is a risk of intra-molecular recognition, which could prohibit its replication. Therefore, replicase-replicase recognition might be more problematic than replicase-template recognition [11]. Whether this is a genuine problem for *in vitro *selected ribozyme RNA replicases cannot yet be ascertained as the templates used to study polymerization are still very short, owing to the limited extension of current enzymes [20-22]. Nevertheless, if such an intrinsic bias in template recognition exists – whichever its direction may be – it significantly influences the evolution of strand preference. Related to this point is the possible correlation between the strand preference in single strands (*k*_*SP *_and *k*_*SM*_) and that in double strands (*k*_*DP *_and *k*_*DM*_). In this study, we investigated two extreme cases, namely where there is either perfect correlation or no intrinsic correlation between these strand preferences. In the latter case, we saw that a complete anti-correlation evolves between the strand preference in single strands and that in double strands. Such complete anti-corrleation is, however, implausible: If a molecule uses the same motif to be recognized by a replicase whether it is single-stranded or double-stranded, then there must be some correlation between *k*_*SP *_and *k*_*DP *_and also between *k*_*SM *_and *k*_*DM*_. Thus, a realistic case should be considered to lie somewhere between these two extreme cases. All these complicating factors suggest a possible focus for future work, for which the results of this study can offer a useful reference for comparison.

Evolution of prebiotic replicators dwelling on surfaces have also been investigated by computer simulations [23,24], and as such serve as an interesting parallel for prebiotic reactions on mineral surfaces. These surfaces have been experimentally shown to provide favorable conditions for possible prebiotic chemical reactions [25-27] (see also refs. [28-30], see refs. [10,31,32] for review), and one may well expect such systems to be spatially structured due to finite diffusion (as is the case for many biological systems). Finite diffusion has previously been shown to play an important role for the evolutionary dynamics of prebiotic replicators [23,33]; namely, the spatial pattern formation of populations and dynamics thereof can generate selective forces that are totally unexpected in a well-mixed system, leading to a reversion of the direction of evolution from that expected under the assumption of infinite diffusion (e.g., see also refs. [24,34-37]; cf. ref. [38]). To this general notion the current study adds that the effect of finite diffusion on evolution can be surprisingly complex such that the outcome of evolution depends on the intensity of diffusion in a non-monotonic way.

Finally, a useful theoretical idea obtained from the current study is the explicit consideration of the relative time-scale of local and global dynamics. In the classical theory of group selection [13,15], the time-scale of the dynamics within a trait-group (local dynamics) is separated from that of the dynamics between trait-groups, and the difference between the two time-scales is not explicitly considered. In the current study, the conceptual framework was built up by focusing on the fact that the two time-scales are directly related via the intensity of diffusion. This allowed us to see at once that the advantage of producing replicases [Pr(Σ_PP_|P) in Eq. (14)] and that of producing templates [$k_{\text{M}|\Sigma_{\text{MP}}}$ in Eq. (15)] are also related, because both are a function of diffusion intensity.

The above time-scale argument is also relevant to the idea of the vesicle-level selection in replicator systems, which deserves some mention in this study. The idea here is that replicators are compartmentalized in vesicles (made of, e.g., lipid bilayers), with vesicle-level selection for those replicator systems that promote the reproduction of vesicles [39,40]. Of particular interest here is the fact that, replicase producers in our system only benefit a local population; a system wherein vesicle-level selection is possible would thus provide an interesting avenue for the study of the evolution of strand preference, for obvious reasons. However, we note that the significant effect of vesicle-level selection does not come for free, in that the relative time-scale of within-vesicle dynamics and between-vesicle dynamics must be optimally chosen [41]. An interesting question would thus be whether vesicle-level selection can result in evolution of a greater strand preference than that seen for the finite diffusion system studied here.

## Conclusion

• Even in an early cell-free phase of evolution, a system of self-replicating RNA with strand displacement can evolve strand preference towards the production of replicases if diffusion is finite and the decay rate is sufficiently high.

• The direction of strand preference evolution – either towards producing more replicases (*r *> 1/2) or towards producing more templates (*r *< 1/2) – depends on the intensity of diffusion in a non-monotonic manner. The mechanism underlying this dependence is elucidated by the multi-scale analysis of the replicator dynamics. The crucial parameter conditions that determine this direction have been identified.

• Replicators with extreme values of *r*, representing near exclusive replicase generation (replicase transcribers) or template generation (parasitic genome copiers), can emerge and coexist if diffusion is finite and where rates of copying for a single-stranded (+) or (-) strand template can freely evolve.

• This study provides yet another illustration of the fact that the explicit consideration of spatially extended systems is important for the study of evolution, since this can significantly change the dynamics of evolution through spatial segregation and/or spatial pattern formation of populations.

## Methods

### The details of the CA model without complex formation

We employed the method developed in ref. [19] (with some modification to it) to model the system of Reaction (1). The model consists of a two-dimensional square grid and molecules [P or M or D as in Reaction (1)] located on the grid. One square of the grid can either contain one molecule or be empty [∅ in Reaction (1)]. The size of the grid is 300 × 300 squares, unless otherwise stated. The boundary of the grid is toroidal. The temporal dynamics of the model is run by consecutively applying the following algorithm, which simulates Reaction (1) and diffusion:

1 Randomly choose one square from the grid. Reaction (1) or diffusion event can take place in this square. The set of possible events that can take plance in this square is determined by the content of the square. If a square is empty (∅), only diffusion can occur. If a square contains a molecule, then the possible reactions are the replication reaction(s) where the molecule is a template (rather than a replicase) and the decay reaction. (For example, if a molecule is P, then either diffusion or $2\text{P}\overset{k_{SP}}{\underset{}{\to }}\text{P}+\text{D}$ or $\text{P}\overset{d}{\underset{}{\to }}\emptyset$ can happen.)

2 Choose an event from the possible events according to the probabilities of the events. If an event is diffusion, its probability is proportional to Δ. If an event is a reaction, then its probability is proportional to its rate constants (viz. *k*_*SP*_, *k*_*SM*_, *k*_*DP*_, *k*_*DM *_and *d*), which are taken from the molecule in the chosen square. A common proportionality coefficient (denoted *c*) is multiplied to these rates to calculate the probabilities for all possible events. The value of *c *sets the time-scale of the system, and is arbitrarily chosen so as to keep the sum of probabilities relevant in this step of the algorithm below 1.

3 a If the chosen event is of first order (viz. decay reaction), it simply takes place. If decay takes place, the molecule in the square is removed from the system.

b If the chosen event is of second order (viz. diffusion and the replication reaction of single strands), choose a square randomly from the eight squares adjacent to the chosen square (Moore neighbors). For a reaction, if the square chosen second contains a replicase (P), then the replication happens such that the molecule of the square chosen first is converted to the double-stranded form (D). If the square chosen second does not contain the right molecule, nothing happens. For diffusion, whatever the contents of the square chosen second is, the contents of the two squares are swapped.

b If the chosen event is of third order (viz. the replication reaction of a double-strand molecule, i.e. strand displacement), choose two different squares randomly from the Moore neighbors. If one of the squares contains a replicase (P) and the other is empty (the order of choice does not matter), the strand displacement happens such that a new molecule – which is a copy of the molecule in the square chosen first (either in the form of P or M depending on the type of reactions) – is produced in the empty square. Upon the production of a new molecule, a mutation can occur in the newly produced molecule as described before (see "The Cellular Automata model" under Results). If the squares chosen second and third do not contain the right types of molecules, nothing happens.

One time-step of a simulation is arbitrarily defined as the consecutive application of the above algorithm to the system by *N *times where *N *equals to the total number of squares in the grid (thus, on average, each square is chosen once in one time-step).

As described previously [19], the above model is closely related to the Gillespie algorithm [14]. In particular, in the limit of Δ → ∞, the dynamics of the above CA model becomes the same as that of the Gillespie algorithm version of the current system with the same parameters (which must be properly scaled).

Although the limit Δ → ∞ cannot, of course, be taken in actual computation, a simple modification to the above algorithm makes it possible to simulate the case of Δ = ∞: For higher order reactions, the modified algorithm chooses a secondary (and tertiary) square from the whole grid (without choosing the same square twice) instead of from the Moore neighbors of the square chosen first.

With regard to the method of mutations, we note that the particular implementation adopted here does not make any qualitative difference in the results; e.g. when *r *is chosen from a uniform distribution in [0,1] upon mutation, essentially the same results are obtained with the same mutation rate, *μ *= 0.01 (data not shown).

The above algorithm was implemented with the help of CASH library [42]. The source code is available upon request.

### Measurements of *n*_intra _and *n*_inter_

The set up of the simulations to measure *n*_intra _and *n*_inter _are described under "Multi-scale analysis of the model" in Results. Here, we describe some details of the measurements.

*n*_intra _denotes the number of molecules of the same species (kind) in the Moore neighborhood of one molecule; i.e. the number of molecules of the same kind that a given molecule "meets". Similarly, *n*_inter _is the number of molecules of the other species a molecule meets. ${\bar{n}}_{\text{intra}}$ and ${\bar{n}}_{\text{inter}}$ are the average taken over all individuals of a species, which is arbitrarily chosen from the two species [this choice does not affect the measurement of ${\bar{n}}_{\text{intra}}$ and ${\bar{n}}_{\text{inter}}$ because of the symmetry in the simulation set up (see "Multi-scale analysis of the model" under Results)].

*n*_intra _and *n*_inter _depend on the population density of the species an individual is observing: for *n*_intra_, the density of the same species; for *n*_inter_, the density of the other species (density is calculated as the number of individuals divided by the total number of squares in the grid). Hence, ${\bar{n}}_{\text{intra}}$ and ${\bar{n}}_{\text{inter}}$ are measured as a function of the density of the observed species. In order to obtain data from many regions of possible density, a simulation is initialized with various frequency compositions of the two species. Then, ${\bar{n}}_{\text{intra}}$ and ${\bar{n}}_{\text{inter}}$ are periodically measured for a given time-step interval, which is taken to be large enough to avoid any apparent correlation between successive measurements.

### The ODE model with complex formation

The ODE model with one species is formulated as follows:

(18) $$\begin{array}{c} \dot{P}=-k_{SP}P^{2}-(k_{DP}+k_{DM})DP+b(C_{P}+C_{M})+\kappa \theta (C_{P}+2C_{M})-dP,\\ \dot{M}=-k_{SM}PM+\kappa \theta C_{P}-dM,\\ \dot{D}=(k_{SP}P+k_{SM}M)P-(k_{DP}+k_{DM})DP+b(C_{P}+C_{M})+\kappa \theta (C_{P}+C_{M})-dD,\\ {\dot{C}}_{P}=k_{DP}PD-bC_{P}-\kappa \theta C_{P}-dC_{P},\\ {\dot{C}}_{M}=k_{DM}PD-bC_{M}-\kappa \theta C_{M}-dC_{M}, \end{array}$$

where *θ *= 1 - *P *- *M *- *D *- *C*_*P *_- *C*_*M *_; *P*, *M *and *D *have the same meaning as in Eq. (2); and *C*_*P *_(resp. *C*_*M*_) denotes the concentration of C_P _(resp. C_M_) in Reaction (17).

The ODE model with two species is formulated by assuming that replicases do not discriminate between templates. The model involves 14 variables, namely *P*_*i*_, *M*_*i*_, *D*_*i*_, *C*_*P*;*i*,*j*_,*C*_*M*;*i*,*j*_, where *i *and *j *denote the species (*i *= 1 or 2; *j *= 1 or 2); and *C*_*P*;*i*,*j *_(resp. *C*_*M*;*i*,*j*_) denotes the concentration of C_P _(resp. C_M_) that is formed between D of species *i *and P of species *j*. Since the equations are lengthy, they are described in Additional file 1.

Numerical integration was performed by using GRIND [43].

### The details of the CA model with complex formation

The CA model with complex formation is an extension of the model without complex formation. A complexed molecule (C_P _or C_M _) is represented by one molecule of P and one molecule of D that are located in two contiguous squares. The details of the reaction-diffusion algorithm are very similar to that of the model without complex formation (except for diffusion and some other minor differences, which are described below).

Diffusion is implemented by swapping the position of molecules. However, since a complex molecule must occupy two contiguous squares, the swapping must be done such that it does not break this requirement. There are three possible cases of swapping: (1) one involving no complex molecule; (2) one involving one complex molecule; (3) one involving two complex molecules. In case (1), swapping is done as before. In case (2), let *x *and *y *be the squares chosen for diffusion (which is chosen first does not matter), and let us suppose that *x *contains a non-complex molecule or is empty, and *y *contains a complex molecule. Let *y' *be the square in which the other molecule of the complex in *y *is located (thus, *y *and *y' *are adjacent to each other). Then, swapping is done in the following manner: *x *→ *y'*; *y *→ *x*; *y' *→ *y*, where arrows mean that the content of the left square moves to the right square. In case (3), let us suppose that *x *also contains a complex, and, similarly, let *x' *be the square in which the other molecule of the complex in *x *is located. Then, swapping is done as follows: *x *→ *y'*; *x' *→ *y*; *y *→ *x'*; *y' *→ *x*.

Reaction events are implemented by the straightforward extension of the algorithm described under "The details of the CA model without complex formation" (see Methods). Some minor differences are that when the square chosen first contains a complex molecule, then a secondary square is chosen from the seven squares adjacent to the first square, excluding the square that contains the other molecule of the complex (i.e. the molecule which the molecule chosen first is making a complex with, which is always located in the neighborhood of the molecule chosen first). Another difference is that when the square chosen first is empty, a strand displacement reaction can occur if the randomly chosen neighbor is a complexed molecule. Thus, in the system with complex formation strand displacement can happen whether a complex molecule is chosen first or an empty square is chosen first (note that in the system without complex formation a double-stranded molecule must be chosen first for strand displacement to happen). This effiectively doubles the rate represented by *κ *(see also the next paragraph).

Finally, we note that since one complex molecule occupies two squares, the probability that a complex molecule is picked up in the process of choosing the first square in the  reaction-diffusion algorithm is twice that of a non-complex molecule. This can make the rate of decay and the intensity of diffusion effectively double. This is compensated simply by halving each parameter for complex molecules. The other event that involves complex molecules is strand displacement, and for this, the doubling of the rate (*κ*) is not compensated. This again effectively doubles the rate represented by *κ*. Combined with the doubling described in the previous paragraph, the rate represented by *κ *is thus quadrupled. However, this is unimportant, for it simply means that when the CA model with complex formation is quantitatively compared to the ODE model with complex formation, the value of *κ *in the CA model must be a quarter of that of the ODE model.

## Reviewers' comments

### Reviewer's report 1

Eugene V Koonin, National Center for Biotechnology Information, National Library of Medicine, National Institutes of Health.

This is another in the series of excellent modeling studies from the Hogeweg group where inclusion of compartmentalization as an explicit parameter in a mathematical model of genome replication leads to non-trivial results. In this case, the result is the emergence of a symmetry favoring the formation of either the plus-strand (replicase) or the minus strand (template). There sults, i.e., the preferential production of either plus or minus strands, strongly depend on diffusion intensity emphasizing the emergence of complex behavior in simple models. These results are definitely relevant for our understanding of the range of possibilities of primordial replicator systems.

#### Authors' response

We thank Dr. Koonin for his comments, and like to merely add that "inclusion of compartmentalization" here means that diffusion is finite rather than compartmentalization by, for example, vesicles.

### Reviewer's report 2

*Rob Knight, University of Colorado*.

In this paper, Takeuchi *et al. *introduce a simple but powerful model of strand-specific replication based on differential equations. Although many studies of replicators, dating at least back to Eigen's work in the 1970s, have used related techniques, this paper goes beyond them by introducing strand specificity into the model and by explicitly incorporating spatial considerations such as diffusion using a cellular automata framework. The model shows several interesting phenomena, e.g. "speciation" into two populations of replicators under some conditions, and clear evolution of strand preference and evolution of parasitism under others.

I believe the manuscript is suitable for publication as-is. It raises many interesting questions: for example, we know that real polymerases donot incorporate the four nucleotides with equal efficiency, so could strand bias be seeded by small differences in composition between the strands? Similarly, the results presented do not allow for competition based on length, which is an important feature of many related models. However, these are topics for later research and need not be addressed in the present work.

#### Authors' response

We thank Dr. Kinght for his comments on our manuscript.

### Reviewer's report 3

István Scheuring, Loránd Eötvös University (nominated by David H Ardel, University of California, Merced).

Report: The evolution of self-replicating RNA molecules is one of the central problem of early evolution. The complexity of this phenomenon has been studied from different point of view, but the problem of emergence of double-strande dmolecules is generally neglected. This present work uses a detailed model for the dynamics and evolution of RNA replication, assuming that double stranded RNA can be copied by strand displacement. The authors have studied whether strand preference can be evolved in this dynamical system, and found that limited dispersal may have a crucial role in maintaining this preference in spatially explicit models. They showed that the coexistence of replicase producers and template producers is possible under sufficient conditions. This specialization can be considered as an ancient form of transcription.

I have several questions and suggestions connected with the model:

They assumed that decay rate of the single and the double strands are the same. This simplification makes the analysis tractable, but it is chemically improbable. It is more plausible to assume that decay rate of the double strand is much smaller than decay rate of single strands. The question whether the main conclusions remain the same with this assumption?

#### Authors' response

*This is a good point. Double-stranded molecules would indeed be expected to have slower decay rates than single-stranded molecules *[17], *and this may be an important factor in the evolutionary dynamics: On the one hand, decreasing the decay rate of double-strande dmolecules (D) would decrease the advantage of producing single-stranded (+) molecules (P), because decreasing the decay rate increases the mixing effect of diffusion (as explained in main text). On the other hand, decreasing the decay rate of D would also decrease the advantage of producing single-stranded (-) molecules (M). This can be seen from the fact that decreasing the decay rate of D decreases the net production rate of D at steady state, and that the advantage of producing M lies in this reaction.*

*We have now examined the case of smaller decay rates of D. We reduced the decay rate of D by a factor of ten relative to that of S and M. The results of simulations (without complex formation) are shown in Fig. 11 of Additional file *1, *where *$\bar{r}$*is plotted as a function of *Δ. *As Fig. 11 shows, the behavior of *$\bar{r}$*as a function of *Δ *is qualitatively the same as before although the quantitative difference can be quite large for smaller diffusion rates. The value of *$\bar{r}$*for d *= 0.01 *sharply increases between *Δ = 0.03 *and *1 *(note that this is also observed in *Fig. 3). *This observation is consistent with our explanation that the decay rate does not change the point at which the advantage of producing M critically decreases. Moreover, Fig. 11 shows that the value of *Δ *for which *$\bar{r}$*takes a minimum for d *= 0.01 *is shifted to a slightly lower value. In addition, *$\bar{r}$*now shows slightly non-monotonic behavior for d *= 0.05. *These observations are also consistent with our previous explanation that the non-monotonicity is caused by the different sensitivity to *Δ *of the two opposing advantages. Finally, the results show that the new value of *$\bar{r}$*is in general smaller than the previous value of *$\bar{r}$. *This indicates that decreasing the decay rate of D has a greater decreasing effect on the advantage of producing P than on that of producing M*

They assume in eq. (4) that the fast process is the production of D. Why is it true? My intuition is that rather the production of M and P is fast compared to production of D.

#### Authors' response

We agree with the reviewer that the production of D would not be fast enough to allow quasi-steady state approximation. Nevertheless, we had assumed the quasi-steady state in dD/dt in order to analyze the dynamics at equilibrium. Since this analysis does not explore the possibility of non-stationary solutions (such as a limit cycle), we had checked our analysis by the numerical integration of Eq.(6) with two species. The results were that no non-stationary solution was observed, and that the conclusion drawn under the quasi-steady state assumption was in agreement with that drawn from the numerical integration. We now mention these points in the text.

I like the 'multi-scale' analysis used in the paper, and generally the effort to explain the results. I have only one minor problem here: More explanation is needed for (8) and (9), and I think (8) and (10) are inconsistent (or I misunderstood something).

#### Authors' response

*We have now added more explanation for Eqs. (8) and (9). On the suggested inconsistency, please note that Eqs.(8) and (10) denote different quantities: While Eq.(8) denotes the probability that a replication reaction happens in a subsystem *Σ_PP_, *Eq. (10) denotes the probability that a replication reaction happens for one molecule of P in *Σ_PP_. *The latter quantity can be obtained by dividing the former by 2, because there are two P molecules that can be replicated in *Σ_PP_, *and because the replication of each molecule is mutually exclusive. The resulting expression can be further simplified by ignoring d. The final result is the RHS of Eq.(10).*

When they studied the evolution of *k*_*SM *_and *k*_*SP*_, they use two general trade-offs for the kinetic constants that is *k*_*SP *_= *k*_*DP*_, *k*_*SM *_= *k*_*DM *_and *k*_*SM *_+ *k*_*SP *_= *k*_*DP *_+ *k*_*DM*_. Both of them seem to be a bit odd, since it is assumed that replication of single strands are as fast as replication by strand displacement. Since it is improbable, I would suggest to modify these assumptions by *αk*_*SM *_= *k*_*DM*_, *αk*_*SP *_= *k*_*DP *_and similarly *α*(*k*_*SM *_+ *k*_*SP*_) = *k*_*DP *_+ *k*_*DM*_, where *α *is a positive constant (probably smaller than 1). The question whether the general conclusions of this section remain valid with this generalization, or not?

#### Authors' response

We thank Dr. Scheuring for his insightful comments and suggestions. We have now examined the case he suggests.

*Unfortunately, there was an unintentional inconsistency in the use of parameters in the original manuscript (for the model without complex formation). In the section where k*_*SM *_*and k*_*SP *_*are kept constant, the parameters were set such that k*_*SM *_= *k*_*SP *_= *k*_*DP *_+ *k*_*DM *_*[i.e.*0.5(*k*_*SM *_+ *k*_*SP*_) = *k*_*DP *_+ *k*_*DM*_*]. However, in the section where k*_*SM *_*and k*_*SP *_*are allowed to evolve, the parameters were set such that k*_*SP *_= *k*_*DP *_*and k*_*SM *_= *k*_*DM *_*or k*_*SM *_+ *k*_*SP *_= *k*_*DP *_+ *k*_*DM*_. *To correct this inconsistency, we re-ran the simulations with the following parameter settings: *0.5*k*_*SP *_= *k*_*DP *_*and *0.5*k*_*SM *_= *k*_*DM*_*, and *0.5(*k*_*SM *_+ *k*_*SP*_) = *k*_*DP *_+ *k*_*DM*_. *The results showed that our general conclusions remain valid undre these parameters. The paper now reports the results that were obtained with these corrected parameters.*

*Additionally, these extra simulations lead to a new insight: there is non-linearity in the anti-correlation between the strand preference in single strands and that in double strands that evolves as a result of diverging selection under finite diffusion. We now mention this result in main text and give details in Fig. 12 of Additional file *1.

By introducing the complex formation into the reaction dynamics the template bias dependence on diffusion is different qualitatively than it is experienced in the simpler model. The question is when the more complex model (17) can be simplified by mode l(1). For example *κ *dependence of the behaviour of the model is interesting.

#### Authors' response

*If κ is increased, the time a molecule spends in the complexed form will become shorter relative to the lifetime of the molecule. Therefore, one can expect that when κ is increased to infinity, the dynamics of reaction (17) would approach that of reaction (1) in some aspects. A previous study has shown that this is indeed the case in a simpler, well-mixed replicator system (i.e. the disadvantage of being replicases due to complex formation diminishes as κ increases) *[19]. *We expect that a similar result holds in the current system too. However, for that, κ might have to be set to an unrealistically high value (according to the previous study, κ might have to be several orders of magnitude greater than k*_*DP *_*and b). Moreover, the result might not hold in a spatial system.*

Comments: Association dissociation of P M and D is not considered in the model.

#### Authors' response

This is correct – we ignored this for simplicity. An important assumption we make is that the association-dissociation reactions might be intrinsically so slow that replicators might employ a replication mechanism that can bypass these reactions, i.e. strand displacement. Given this context, it seems relevant to ignore the association-dissociation reactions, and has the advantage of making the system more tractable.

Substrate can be added for every reaction in (1) and in (17).

#### Authors' response

*The following assumptions about substrates are made in our models. In the Cellular Automaton model, which is the main model we have studied, substrates (nucleotide monomers) were not explicitly considered for simplicity. We instead implemented growth limitation by the limitation of space (the grid size is finite), since this is the simplest way to obtain growth limitation in this model formalism. The space limitation is thus used as a proxy for a finite supply of resources in general (the word "substrate" in the previous manuscript was perhaps misleading in this regard and is now replaced by "resource" in an attempt to make this distinction clearer). Since the reaction *P (or M) → D *does not require an empty square in the CA model, ∅ is not included for this reaction in 1 and 17. Given this simplification of the CA model, the ODE model was constructed so as to be compatible with it.*

Generally more information is needed in the figure legends. Parameters of the simulations are frequently missing.

#### Authors' response

We have now added additional information in the figure legends.

Legend of Fig. 2: I suspect that *k*_*SP *_+ *k*_*SM *_would be the correct form.

#### Authors' response

*The original form is actually correct. Please see also our response above on the parameter setting for k*_*SP *_*and k*_*SM*_.

Fig. 4 is not important.

#### Authors' response

*We think the comparison between Fig. *4* and Fig. *7* gives an important visual aid for understanding of our explanation for the non-monotonicity of *$\bar{r}$.

p. 16:It is not clear when the speciation of replication forms does occur. I suggest to overwrite this paragraph.

#### Authors' response

*It is not exactly clear for which parameters the speciation-like phenomenon happens. Our previous statement that it happens where the parameters were such that *$\bar{r}$ = 1/2 *in Fig. *3* was not precisely correct, in that additional simulations showed that it occurs in a wider range of parameters than we previously thought (the legend of Fig. *9* now mentions the parameters for which the speciation-like phenomenon was observed). The paragraph on speciation (under the section, "The effect of biases in the replication rates of single strands") has been updated to reflect this.*

## Competing interests

The authors declare that they have no competing interests.

## Authors' contributions

AMP initiated the study. NT designed the models. NT and LS implemented the models, ran the simulations andanalyzed the data. NT, PH and LS interpreted the results. NT developed the theoretical framework to understand the results and wrote the first draft of the manuscript, under the supervision of PH. NT, AMP and PH revised the manuscript. All authors read and approved the final manuscript.

## Supplementary Material

Additional file 1 1 Figure 11. This figure shows the results of additional simulations where the decay rate of double-stranded molecules is reduced by a factor of 10 relative to that of single-stranded molecules. The figure is referred to in the Authors' response to Reviewer's report 3.
2 Figure 12. This figure shows the additional results that reveal a non-linear anti-correlation that evolves between *k*_*SP *_and *k*_*DP *_as a result of diverging selection under finite diffusion. This figure is referred to in the section, ''The effect of biases in the replication rates of single strands (a case where *k*_*SP *_≠ *k*_*SM*_')".
3 The description of the ODE model with complex formation for a system of two replicator species. Click here for file
