# Supplementary material for: The evolution of strand preference in simulated RNA replicators with strand displacement: Implications for the origin of transcription
Source: Biol Direct. 2008 Aug 11;3:33. doi: 10.1186/1745-6150-3-33 (PMC2648946; doi:10.1186/1745-6150-3-33)
Supplement: Additional file 1 — 1 Figure 11. This figure shows the results of additional simulations where the decay rate of double-stranded molecules is reduced by a factor of 10 relative to that of single-stranded molecules. The figure is referred to in the Authors' response to Reviewer's report 3. 2 Figure 12. This figure shows the additional results that reveal a non-linear anti-correlation that evolves between kSP and kDP as a result of diverging selection under finite diffusion. This figure is referred to in the section, ''The effect of biases in the replication rates of single strands (a case where kSP ≠ kSM')". 3 The description of the ODE model with complex formation for a system of two replicator species. [file 1745-6150-3-33-S1.pdf]

Additional file 1
(The evolution of strand preference in simulated RNA replicators with strand displacement: implications for the origin of transcription)

N. Takeuchi, L. Salazar, A.M. Poole and P. Hogeweg

1 Figure 11

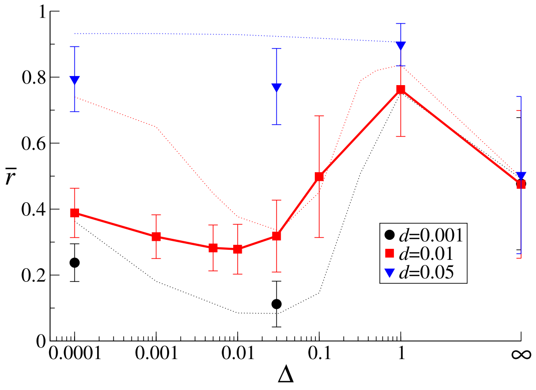

Figure 11: The effect of a smaller decay rate of D.

This figure shows a similar plot to that of Fig. 3 (the coordinate is the population mean of r; the abscissa is the intensity of diffusion; error bars are the mean absolute deviation of r in a population). The plots with error bars represent the results of simulations where the decay rate of D is set to $0.1d$, where d is the decay rate of S and M. The value of d is shown in the graph. The other parameters are identical to those in Fig. 3: $k_{SP} = k_{SM} = k_{DP} + k_{DM} = 1$; $\mu = 0.01$; $\delta_r = 0.1$. For the sake of comparison, the data from Fig. 3 are also shown by the dotted lines (the colors correspond to the values of d).

As seen from this figure, $\bar{r}$ still displays a non-monotonic behavior as a function of Δ with the reduced decay rate for D. Moreover, the sharp increase of $\bar{r}$ between $\Delta = 0.032$ and 1 is also compatible with our explanation that the decrease of the advantage of producing M is invariant with respect to the decay rate. For more explanation regarding this figure, see Authors' response to Reviewer's report 3.

2 Figure 12

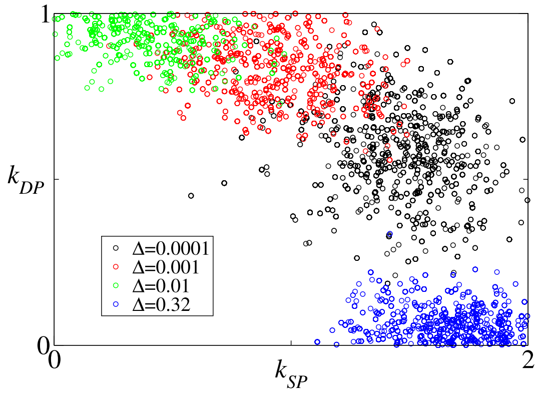

Figure 12: Non-linearity in the anti-correlation between k_{SP} and k_{DP}.

The graph shows a scattered plot of k_{SP} and k_{DP} of every individual in a system at a given time step after the system reached equilibrium, for various values of Δ. The data were obtained from simulations where k_{SP} and k_{SM} were allowed to evolve ($k_{SP} + k_{SM}$ was kept constant; no correlation was presumed between k_{SP} and k_{DP}). The value of Δ is shown in the graph. The other parameters are as follows: $0.5(k_{SP}+k_{SM}) = k_{DP}+k_{DM} = 1$; $d = 0.001$; $\mu = 0.01$; $\delta_r = 0.1$. The mutation of k_{SM} and k_{SP} was implemented in the same way as that of k_{DM} and k_{DP}.

This figure shows that the relationship that evolves between k_{SP} and k_{DP} is non-linear—namely, k_{DP} as a function of k_{SP} is convex. An explanation for this non-linearity can be given as follows. Single-stranded (+) molecules have an replication disadvantage relative to single-stranded (−) molecules (as shown in Fig. 4 in main text). This disadvantage can be compensated by increasing k_{SP} (at the cost of decreasing k_{SM}). This generates a selective force that can cause the non-linearity in question.

3 The ODE model with complex formation

The following is the ODE model with complex formation for a system of two replicator species explained under Methods in main text:

$$
\begin{aligned}
\dot{P}_1 =& \kappa\theta(2C_{M;1,1} + C_{M;1,2} + C_{M;2,1} + C_{P;1,1} + C_{P;2,1}) + b(C_{M;1,1} + C_{M;2,1} + C_{P;1,1} + C_{P;2,1}) \\
& - P_1(k_{SP1}(P_1 + P_2) + D_1(k_{DM1} + k_{DP1}) + D_2(k_{DM2} + k_{DP2})) - dP_1 \\
\dot{P}_2 =& \kappa\theta(2C_{M;2,2} + C_{M;2,1} + C_{M;1,2} + C_{P;1,2} + C_{P;2,2}) + b(C_{M;2,2} + C_{M;1,2} + C_{P;2,2} + C_{P;1,2}) \\
& - P_2(k_{SP2}(P_1 + P_2) + D_1(k_{DM1} + k_{DP1}) + D_2(k_{DM2} + k_{DP2})) - dP_2 \\
\dot{M}_1 =& \kappa\theta(C_{P;1,1} + C_{P;1,2}) - M_1 k_{SM1}(P_1 + P_2) - dM_1 \\
\dot{M}_2 =& \kappa\theta(C_{P;2,2} + C_{P;2,1}) - M_2 k_{SM2}(P_1 + P_2) - dM_2 \\
\dot{D}_1 =& P_1 k_{SP1}(P_1 + P_2) + M_1 k_{SM1}(P_1 + P_2) - D_1(P_1 + P_2)(k_{DM1} + k_{DP1}) \\
& + \kappa\theta(C_{P;1,1} + C_{P;1,2} + C_{M;1,1} + C_{M;1,2}) + b(C_{M;1,1} + C_{M;1,2} + C_{P;1,1} + C_{P;1,2}) - dD_1 \\
\dot{D}_2 =& P_2 k_{SP2}(P_1 + P_2) + M_2 k_{SM2}(P_1 + P_2) - D_2(P_1 + P_2)(k_{DM2} + k_{DP2}) \\
& + \kappa\theta(C_{P;2,1} + C_{P;2,2} + C_{M;2,1} + C_{M;2,2}) + b(C_{M;2,2} + C_{M;2,1} + C_{P;2,2} + C_{P;2,1}) - dD_2 \\
\dot{C}_{P;1,1} =& k_{DP1} D_1 P_1 - C_{P;1,1}(\kappa\theta + b) - dC_{P;1,1} \\
\dot{C}_{P;1,2} =& k_{DP1} D_1 P_2 - C_{P;1,2}(\kappa\theta + b) - dC_{P;1,2} \\
\dot{C}_{P;2,1} =& k_{DP2} D_2 P_1 - C_{P;2,1}(\kappa\theta + b) - dC_{P;2,1} \\
\dot{C}_{P;2,2} =& k_{DP2} D_2 P_2 - C_{P;2,2}(\kappa\theta + b) - dC_{P;2,2} \\
\dot{C}_{M;1,1} =& k_{DM1} D_1 P_1 - C_{M;1,1}(\kappa\theta + b) - dC_{M;1,1} \\
\dot{C}_{M;1,2} =& k_{DM1} D_1 P_2 - C_{M;1,2}(\kappa\theta + b) - dC_{M;1,2} \\
\dot{C}_{M;2,1} =& k_{DM2} D_2 P_1 - C_{M;2,1}(\kappa\theta + b) - dC_{M;2,1} \\
\dot{C}_{M;2,2} =& k_{DM2} D_2 P_2 - C_{M;2,2}(\kappa\theta + b) - dC_{M;2,2} \\
\theta =& 1 - \sum_{i=1}^{2}[P_i + M_i + D_i + 2\sum_{j=1}^{2}(C_{P;i,j} + C_{M;i,j})].
\end{aligned}
$$

The subscript i in P_i, M_i, D_i and k_{XYi} (where $X = S$ or D; $Y = P$ or M) denotes the species. For $C_{P;i,j}$ and $C_{M;i,j}$, the subscript P and M denotes the template strand; the subscript i denotes the species of the template (D_i); and the subscript j denotes the species of the replicase (P_i). The factor of 2 appears in front of $C_{P;i,j}$ and $C_{M;i,j}$ in θ in order to take account of the fact that in the CA model one complex molecule occupies two squares. However, whether or not this factor is taken into account does not qualitatively affect the behavior of the ODE model.
